# DNMT3B aggravated renal fibrosis in diabetic kidney disease via activating Wnt/β-catenin signaling pathway

**DOI:** 10.1038/s41598-025-06713-3

**Published:** 2025-07-01

**Authors:** Lingling Qu, Tong Wang, Jing Kong, Xin Wu, Qingxuan Li, Tianhua Long, Xiaomin An, Yuwei Lu, Yao Mu, Yao Ran, Bing Guo, Mingjun Shi

**Affiliations:** 1 Department of Pathophysiology, Guizhou Medical University, Guiyang, 550025 Guizhou China; 2 Department of Pathology, Northwest Women’s and Children’s Hospital, 710003 Xi’an, Shanxi China; 3 Department of Nephrology, Guiyang First People’s Hospital, Guiyang, 550002 Guizhou China

**Keywords:** Diabetic kidney disease (DKD), Wnt/β-catenin signalling pathway, DNA methylation, DNA methyltransferase 3B (DNMT3B), Secreted frizzled-relatd protein 5 (SFRP5), Diseases, Nephrology, Pathogenesis

## Abstract

The incidence of diabetic kidney disease (DKD) has increased rapidly worldwide in recent decades, and DKD is the leading cause of chronic kidney disease. The Wnt/β-catenin pathway is widely recognized as a critical contributor to DKD. However, how this pathway is activated in DKD is still unknown. Recent studies have revealed that epigenetic mechanisms play key roles in DKD. DNA methylation is an epigenetic mechanism that is essential for regulating gene transcription. Here, we demonstrated that reducing the expression of DNMT3B, a DNA methyltransferase, markedly decreased extracellular matrix (ECM) deposition and diabetic renal fibrosis (DRF). Furthermore, we found that DNMT3B activated the Wnt/β-catenin pathway by suppressing SFRP5 expression in HG-induced renal tubular epithelial cells. Mechanistically, we observed that DNMT3B increased the promoter methylation levels of *sfrp5*, which contributed to a decrease in SFRP5 protein expression. Additionally, Pharmacological disruption of DNA methylation (via 5-Aza) and genetic knockdown of DNMT3B suppressed the Wnt/β-catenin pathway, leading to the attenuation of ECM deposition and DRF. Thus, our study provides a novel understanding of the epigenetic regulation of DKD pathogenesis and a new therapeutic strategy for DKD by disrupting the Wnt/β-catenin pathway.

**Supplementary Information:**

The online version contains supplementary material available at 10.1038/s41598-025-06713-3.

## Introduction

The global prevalence of diabetes mellitus (DM) has increased substantially over the past decades. According to International Diabetes Federation (IDF) Diabetes Atlas (10th edition), the number of adult diabetes patients (20–79 years old) worldwide has reached 537 million, with a prevalence rate of approximately 10.5% as of 2021. Among countries, China has the highest number of DM patients globally, with approximately 170 million people affected^1^. At present, almost 40% of these patients develop diabetic kidney disease (DKD)^2^. Diabetic renal fibrosis (DRF) is a major characteristic of DKD and an important process in renal dysfunction. DRF is attributed to the aberrant accumulation of extracellular matrix (ECM) and epithelial‒mesenchymal transition (EMT)^3^. However, the development of DRF involves multiple pathways, and currently the molecular pathogenesis remains insufficiently understood. Therefore, further research on the mechanism of DRF is highly important for elucidating the progression and advancing preventive strategies of DKD disease.

Previous studies have shown that DRF progression is closely related to the activation of the TGF-β/Smad^4^, PI3K/AKT^5^, MAPK^6^Wnt/β-catenin and other signalling pathways. Among them, the Wnt signalling pathway is highly conserved and plays an important role in regulating renal fibrosis^7^ by regulating the expression of various downstream mediators^8^. Thus, Wnt/β-catenin pathway inhibition might be a potential therapeutic target for alleviating renal fibrosis.

Secreted frizzled-related proteins (SFRPs) are considered pivotal endogenous regulators of the Wnt/β-catenin pathway^9^. On the basis of sequence homology, the SFRP family can be divided into two groups: SFRP1, SFRP2, and SFRP5 comprise one group, and SFRP3 and SFRP4 comprise the other^10^. All SFRPs have 3 similar domains: a signal peptide, an N-terminal cysteine-rich domain (CRD) and a C-terminal netrin-like domain (NTR)^11^. A high degree of overlap exists between the CRD domain in SFRP family proteins and in Frizzled (FZD) receptors, resulting in the blockade of the interaction of Wnt proteins with FZD receptors, which regulates the activity of the Wnt pathway^12^. Recently, it was reported that SFRP5 protects against renal fibrosis by inhibiting the Wnt/β-catenin pathway^13^. To date, the regulatory mechanisms of SFRP5 in DRF and the factors that regulate SFRP5 are still unclear.

Many studies have reported that SFRP5 is frequently silenced and correlated with promoter methylation in tumours and inflammation^14–18^, and that demethylating drugs can reverse this silencing to restore SFRP5 expression^19^. DNA methylation mainly occurs at CpG dinucleotides and is catalysed by DNA methyltransferases (DNMTs). In the reaction, the 5’-C of the cytosine ring on the CpG island of a target promoter obtains a methyl group from S-adenosylmethionine, and a DNMT converts it into 5-methylcytosine. DNMTs are pivotal enzymes that catalyse DNA methylation. The human genome encodes five DNMTs: DNMT1, DNMT2, DNMT3A, DNMT3B and DNMT3L. DNMT1, DNMT3A and DNMT3B are canonical cytosine‑5 DNMTs that catalyse the establishment of methylation patterns in genomic DNA. The primary function of DNMT1 is to maintain genome methylation, whereas DNMT3A and DNMT3B predominantly catalyse the de novo methylation of unmethylated or semimethylated DNA. Recent studies have shown that abnormal DNA methylation at the promoters of genes is associated with renal fibrosis in renal injury models^20–22^, indicating that DNA methylation may play an essential role in renal fibrosis and providing a potential therapeutic target for DKD. Emerging evidence suggests DNMT3B (rather than DNMT1 or DNMT3A) as a sensitive methyltransferase in organ fibrosis^23–25^. It was reported that the expression of DNMT3B was significantly upregulated in kidney tissues of CKD patients and different RTEC cell lines induced by TGF-β1^23^. Previously, studies both in vivo and in vitro demonstrated that abnormal DNA methylation induces transcriptional dysregulation of key proteins involved in the ECM remodeling and the sustained fibroblast activation, consistently^26^.However, few studies have been focused on how DNMT3B regulates the mechanisms of diabetic renal fibrosis.

On the basis of these findings, we hypothesized that DNA methylation is associated with DKD and DNMT3B abnormally activates the Wnt/β-catenin signalling pathway by regulating *sfrp5* promoter methylation. We used renal tubular epithelial cells (RTECs) and kidneys from diabetic mice to investigate the contribution of DNMT3B to the abnormal activation of the Wnt/β-catenin signalling pathway and deposition of ECM. Additionally, we used the demethylation drug 5-azacytidine (5-Aza) and shRNA to decrease the expression of DNMT3B to elucidate the specific molecular mechanism by which DNMT3B regulates SFRP5. Through the above experiments, we aimed to explore the relationships among DNMT3B, SFRP5 and Wnt/β-catenin signalling in the renal fibrosis of DKD patients.

## Results

### **High glucose promoted Wnt/β-catenin pathway activation and extracellular matrix formation** in vitro **and** in vivo

**Fig. 1:**
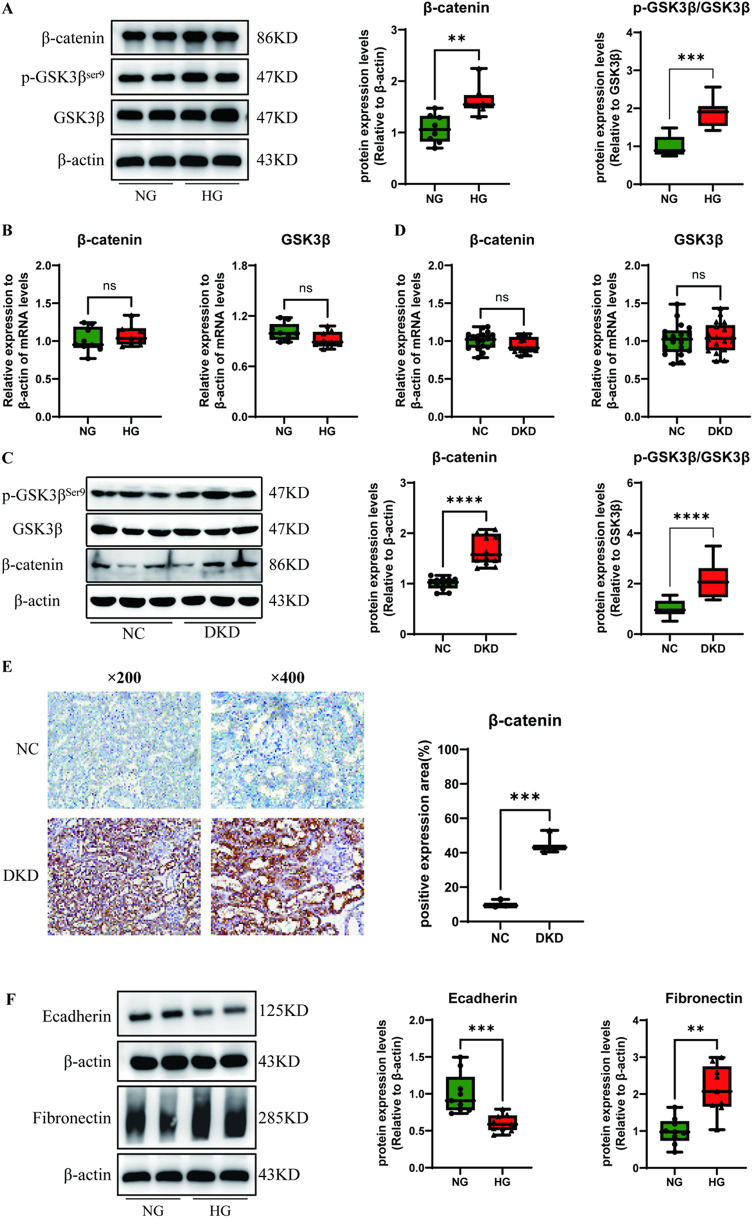

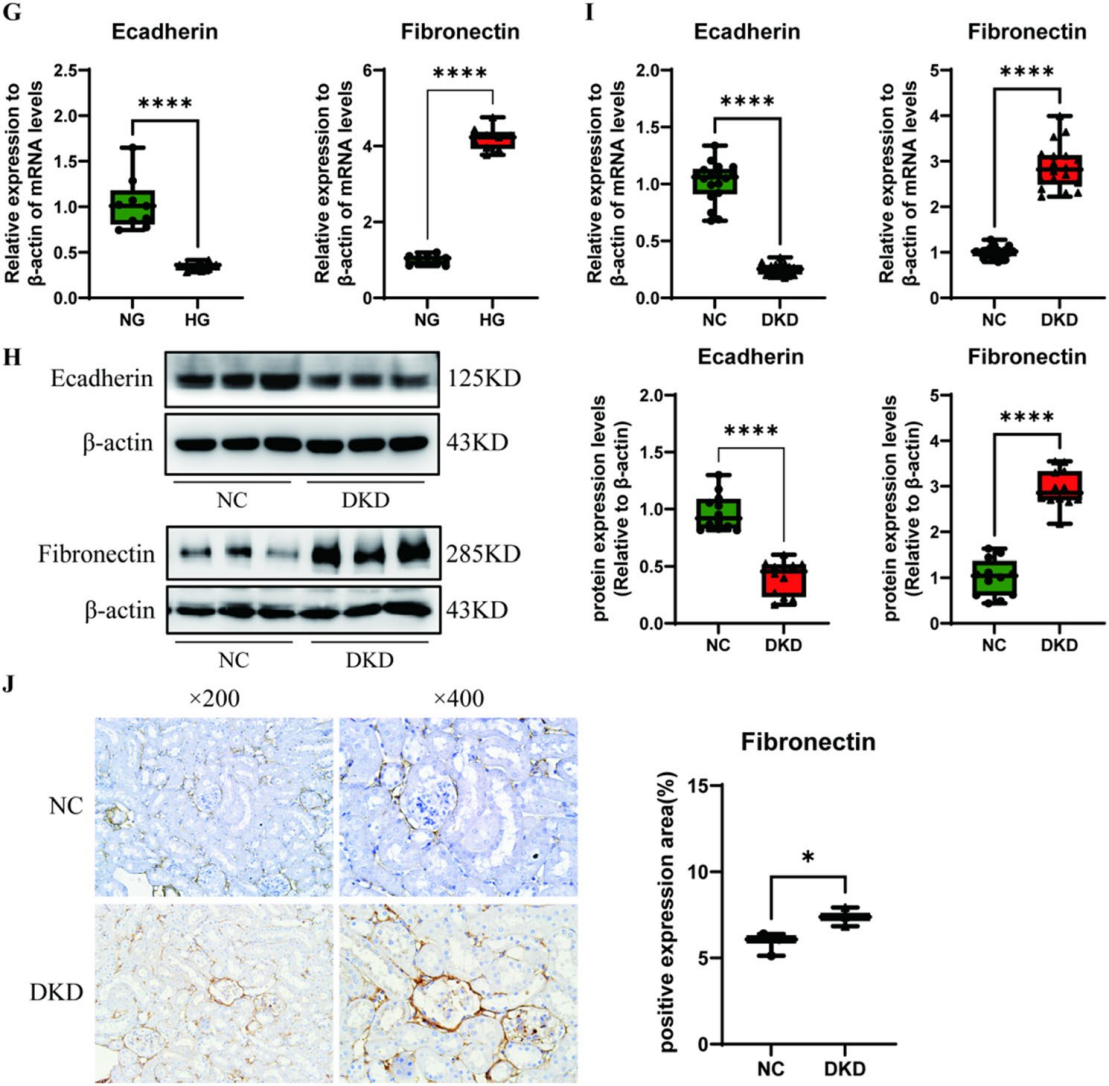
High glucose promoted Wnt/β-catenin pathway activation and extracellular matrix formation in vitro and in vivo. (**A**) Protein and (**B**) mRNA expression of Wnt/β-catenin pathway-related factors were measured via Western blotting and real-time PCR using RTECs after stimulation with high glucose (HG, 30 mM) for 48 h (*n* = 3). (**C**) Protein expression and (**D**) mRNA levels of the above factors were measured in DKD renal tissues from mice (*n* = 6). (**E**) Immunohistochemistry (IHC) was used to assess the expression of β-catenin in DKD renal tissues from mice, and the percent positive area was scored using ImageJ. Fibronectin and E-cadherin (**F**) protein expression and (**G**) mRNA levels were measured via Western blotting and real-time PCR using RTECs after stimulation with high glucose (HG, 30 mM) for 48 h (*n* = 3); (**H**) protein and (**I**) mRNA expression levels of fibronectin and E-cadherin were measured in DKD renal tissues (*n* = 6); (**J**) IHC was used to assess the expression of fibronectin in DKD renal tissues from mice, and the percent positive area was scored using ImageJ. The data are expressed as means ± SDs. DKD, diabetic kidney disease; RTECs, renal tubular epithelial cells. *p < 0.05, **p < 0.01, ***p < 0.001, and ****p < 0.000 vs. control.

To clarify whether the Wnt/β-catenin pathway, the deposition of ECM and EMT are involved in DRF, we used high glucose-stimulated RTECs in vitro and STZ-induced DM mouse kidneys in vivo as DKD models. Previous studies have shown that the nuclear translocation of β-catenin and the phosphorylation of GSK3β at Ser9 are critical factors in the canonical Wnt signalling pathway^27^. As expected, the protein levels of β-catenin and p-GSK3β^ser9^ were higher in both high glucose-stimulated RTECs and DKD renal tissues than in the corresponding negative controls (Fig. 1A and C). However, neither β-catenin nor GSK3β stimulation with high glucose in vivo and in vitro did not clearly alter the mRNA levels compared with those in the normal group (Fig. 1B and D). The immunohistochemistry (IHC) results revealed that the protein expression and nuclear translocation of β-catenin were significantly increased in DKD renal tissues (Fig. 1E).

Moreover, we assessed the expression of factors involved in extracellular matrix deposition. The results revealed that the protein and mRNA levels of fibronectin were markedly increased while E-cadherin were decreased in the DKD models in vivo and in vitro (Fig. 1F-I). Consistent with the previous results, IHC indicated that the expression of fibronectin was increased in DKD renal tissue from mice (Fig. 1J). These data suggest that ECM proteins accumulate and that the Wnt/β-catenin pathway is activated in RTECs after long-term high glucose stimulation both in vitro and in vivo.

### SFRP5 inhibited Wnt/β-catenin signalling in DKD models

**Fig. 2:**
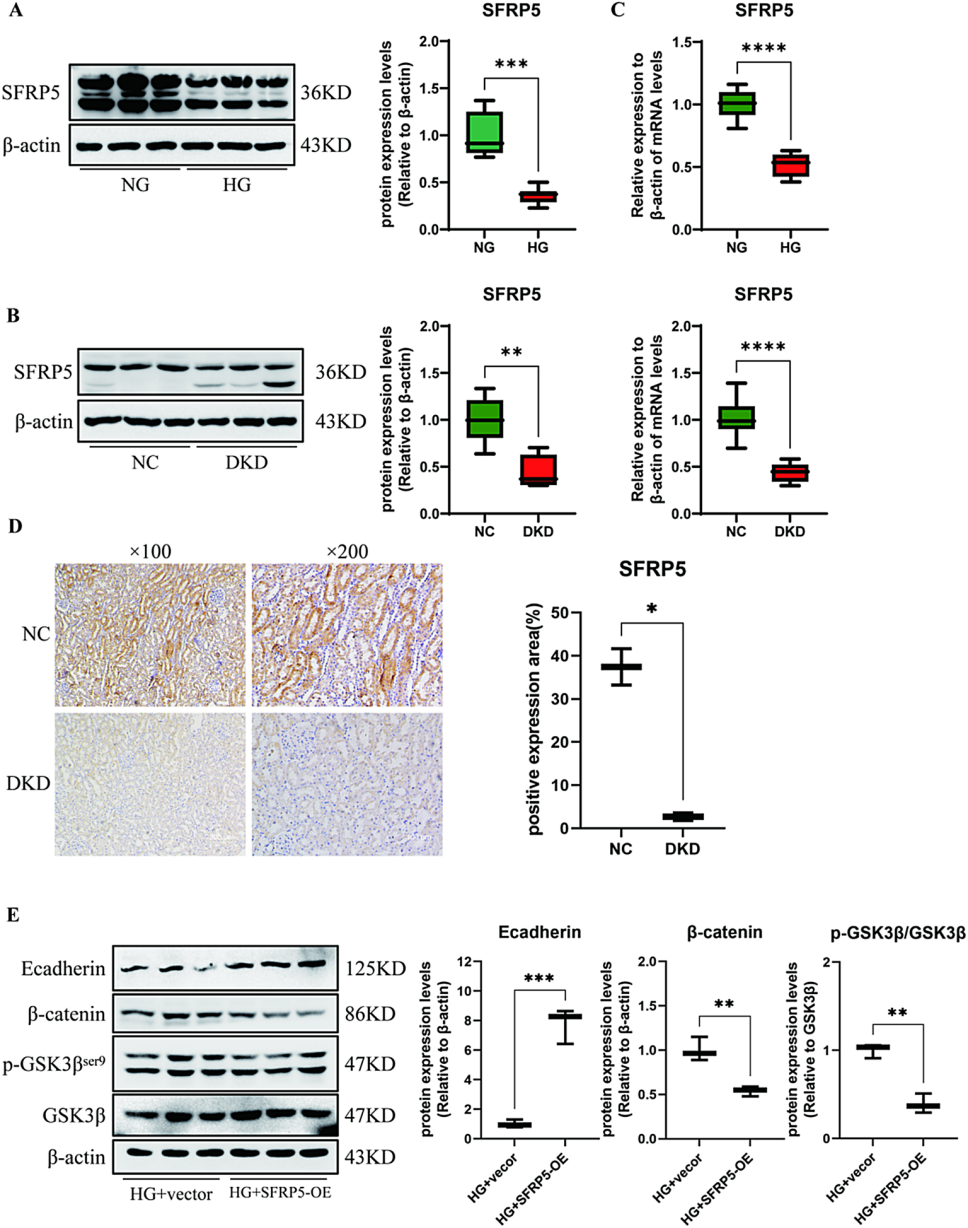
SFRP5 inhibited Wnt/β-catenin signalling in DKD models. (**A**) Protein expression of SFRP5 was measured via Western blotting using RTECs after stimulation with high glucose (HG, 30 mM) for 48 h (*n* = 3). (**B**) Protein expression of SFRP5 was measured via Western blotting using DKD renal tissue from mice (*n* = 6); (**C**) mRNA expression of SFRP5 was measured in HG-stimulated RTECs (*n* = 3) and DKD renal tissues (*n* = 6). (**D**) IHC was used to assess the expression of SFRP5 in DKD renal tissue from mice, and the percent positive area was scored using ImageJ. (**E**) Protein expression of E-cadherin and Wnt/β-catenin pathway-related factors was measured using RTECs after stimulation with HG (*n* = 3). The data are expressed as means ± SDs. DKD, diabetic kidney disease; RTECs, renal tubular epithelial cells. *p < 0.05, **p < 0.01, ***p < 0.001, and ****p < 0.000 vs. control.

SFRP5 is a canonical antagonist of the Wnt signalling pathway. While Wnt signalling plays a significant role in regulating organ fibrosis, it is unclear whether SFRP5 inhibits ECM deposition through this pathway. First, we assessed the protein and mRNA expression of SFRP5 in high glucose-stimulated RTECs and DKD renal tissue from mice. The results revealed that the protein expression of SFRP5 was reduced both in vivo and in vitro DKD models than in the respective control groups (Fig. 2A-C). The IHC results were consistent with the above data for DKD mouse renal tissues (Fig. 2D). Next, we examined the effect of SFRP5 on the Wnt/β-catenin signalling pathway in renal fibrosis. The results revealed that the upregulation of SFRP5 expression decreased the phosphorylation level of GSK3β at Ser9 and the total β-catenin level (Fig. 2E). Taken together, these results indicate that SFRP5 inhibits the development of renal fibrosis by inactivating the Wnt/β-catenin signalling pathway.

### The expression of SFRP5 was decreased after *sfrp5* promoter hypermethylation

**Fig. 3:**
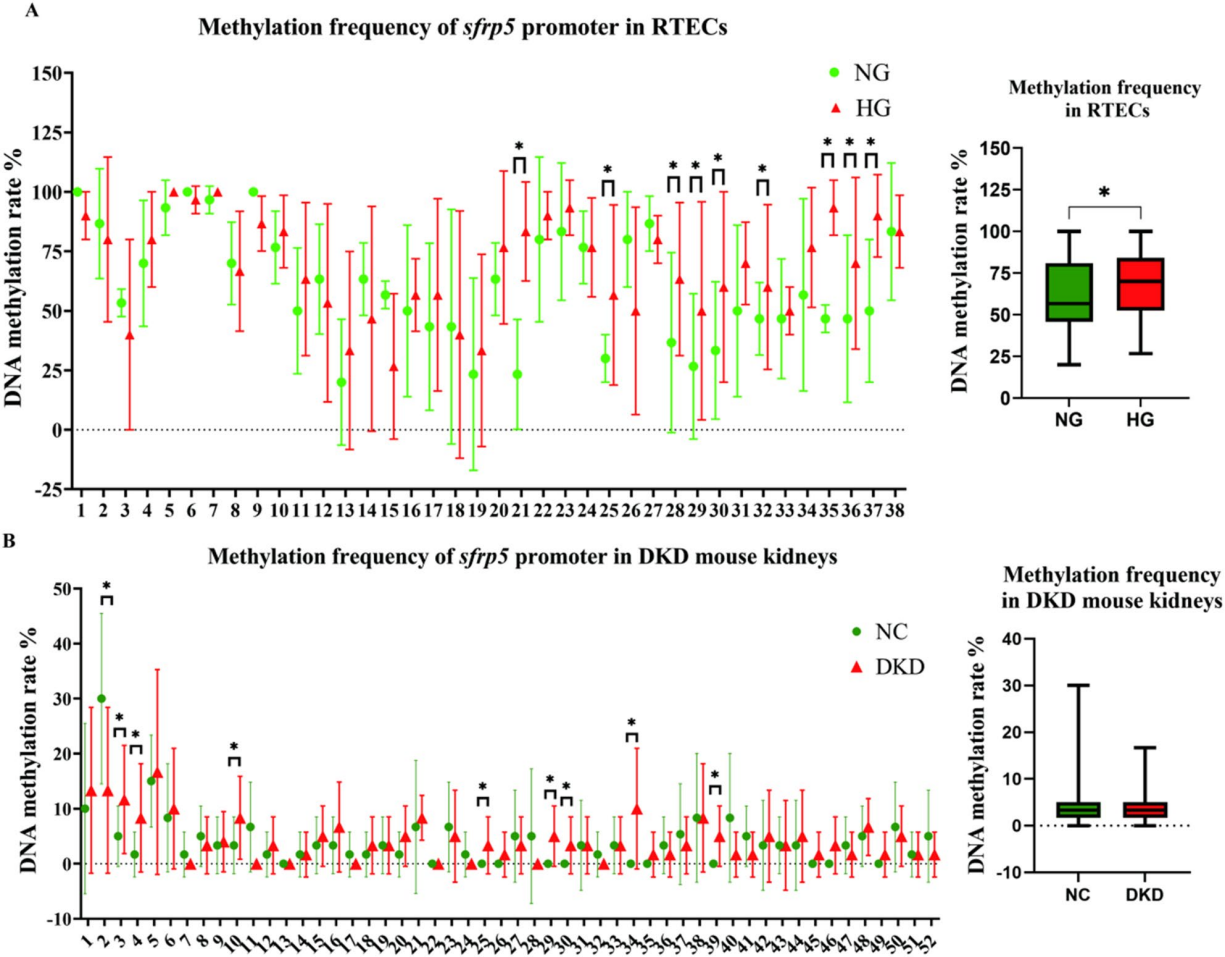
The expression of SFRP5 was decreased after ***sfrp5*** promoter hypermethylation. (**A**) and (**B**) *sfrp5* promoter methylation rates were assessed in high glucose-stimulated RTECs (*n* = 3) and DKD renal tissues from mice (*n* = 6); the data are expressed as means ± SDs. *p < 0.05 vs. control.

Previous studies revealed that the *sfrp5* promoter is highly methylated in a variety of tumours^14–16^. Hence, we hypothesized that high-glucose stimulation suppresses the expression of SFRP5 by catalysing *sfrp5* promoter hypermethylation, which results in *sfrp5* gene silencing in DKD. To support the above hypothesis, we first investigated the methylation status of the *sfrp5* promoter region in normal and DKD renal tissues from mice. The UCSC website was used to obtain the gene sequence and identify the promoter region of *sfrp5*, and Methyl Primer Express v1.0 software was used to design methylation-specific primers. We examined the methylation status of the *sfrp5* promoter region in different samples via bisulfite sequencing (BSP) PCR. The results revealed that *sfrp5* promoter methylation frequency in DKD renal tissue and in high glucose-stimulated RTECs was greater than that in the respective control groups (Fig. 3A-B). Together, these data suggest that high-glucose stimulation increased methylation within the promoter region of *sfrp5* both in vivo and in vitro.

### DNMT3B decreased the expression of SFRP5 while elevated the activation of Wnt/β-catenin signalling and the formation of extracellular matrix in HG-induced RTECs

**Fig. 4:**
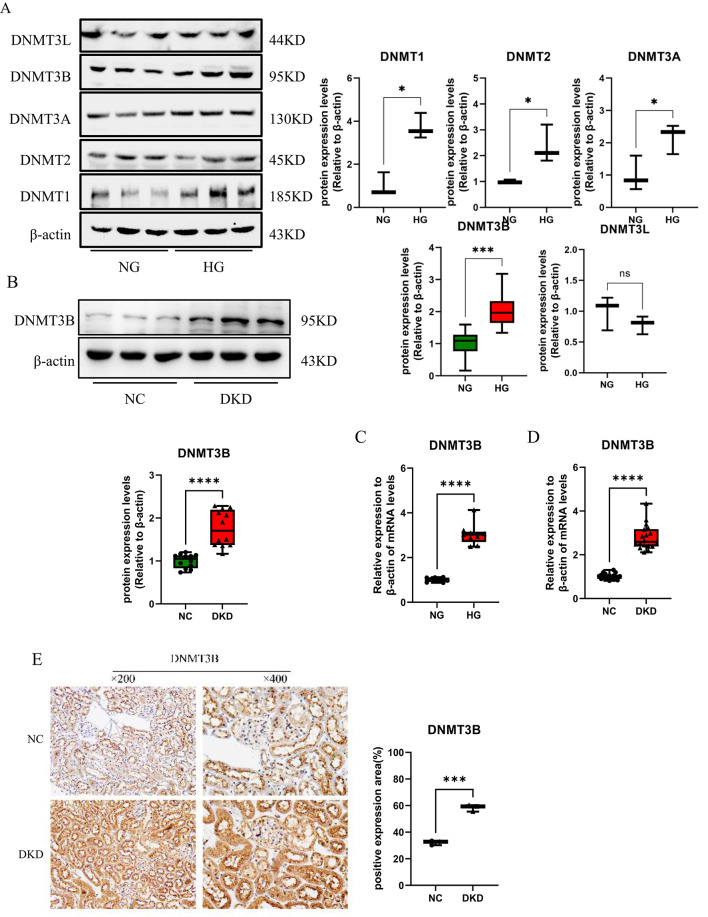

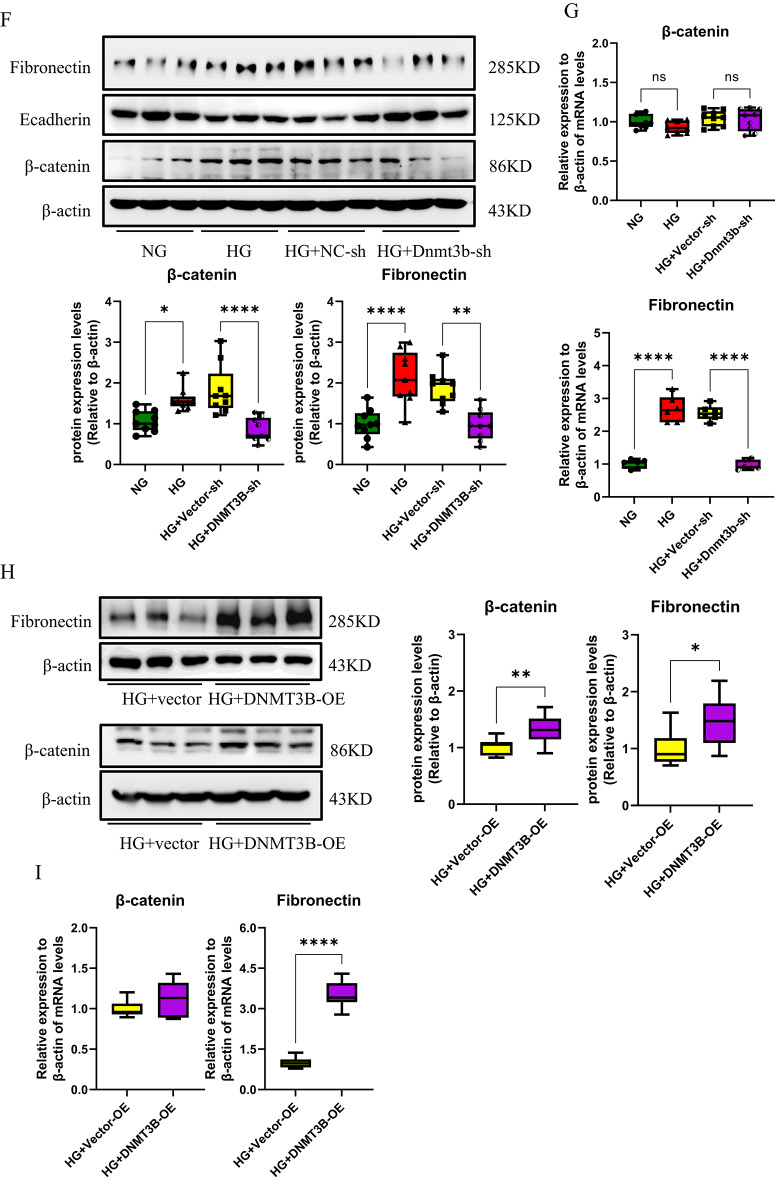

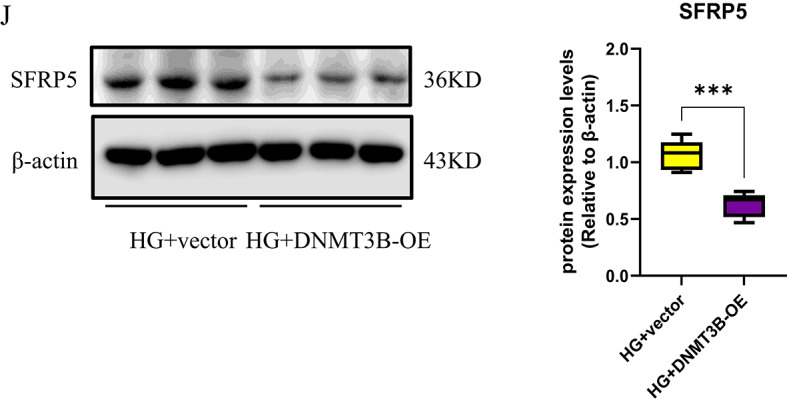
DNMT3B elevated the activation of Wnt/β-catenin signaling and the formation of extracellular matrix in HG-induced RTECs. (**A**) The protein expression of DNMTs was assessed via Western blotting using RTECs after stimulation with high glucose (*n* = 3); (**B**) the protein expression and (**D**) mRNA expression of DNMT3B were assessed in DKD renal tissue from mice (*n* = 6); (**A**) the protein expression and (**C**) mRNA expression of DNMT3B were measured in high glucose (HG)-stimulated RTECs (*n* = 3); (**E**) IHC was used to detect the expression of DNMT3B in DKD renal tissues, and the percent positive area was scored using Image J; (**F**) the protein expression and (**G**) mRNA expression of ECM-related markers (E-cadherin and fibronectin) and β-catenin were assessed via Western blotting and qPCR using high glucose-stimulated RTECs after transfection with or without DNMT3B-shRNA for 48 h; (**H**) the protein expression and (**I**) mRNA expression of ECM-related markers (E-cadherin and fibronectin) and β-catenin were assessed via Western blotting and qPCR using high glucose-stimulated RTECs after transfection with or without DNMT3B-OE plasmid. (**J**) the protein expression of SFRP5 were assessed via Western blotting using high glucose-stimulated RTECs after transfection with or without DNMT3B–OE plasmid for 48 h; The data are expressed as means ± SDs. RTECs, renal tubular epithelial cells. *p < 0.05, **p < 0.01, ***p < 0.001, and ****p < 0.000 vs. control.

To further identify the specific DNMTs regulating the *sfrp5* gene promoter, we explored the diverse expression patterns of DNMTs in high glucose-stimulated RTECs. The results revealed that compared with those in normal samples, the protein levels of DNMT1, DNMT2, DNMT3A and DNMT3B were higher and the level of DNMT3L was not obviously abnormal in RTECs stimulated with high glucose; notably, there was a significant difference in the level of DNMT3B than other DNMTs (Fig. 4A). The above findings suggested that DNMT3B, the expression of which was higher than that of other DNMTs, was activated in RTECs cultured under high glucose conditions. Subsequent analysis indicated that the protein expression of DNMT3B was elevated in DKD mouse renal tissues corroborated by Western blot and IHC, compared to controls(Fig. 4B and E). Furthermore, the DNMT3B mRNA levels were significantly higher than their respective controls in both HG-stimulated RTECs and DKD renal tissues (Fig. 4C-D).

To investigate the potential roles of DNMT3B in DKD, we analysed whether the activation of Wnt/β-catenin pathway was altered by DNMT3B expression. The results showed that the protein levels of β-catenin and fibronectin were decreased while the E-cadherin levels were increased after DNMT3B knockdown used by shRNA in HG-cultured RTECs, compared to HG group (Figure 4F). Corresponding mRNA analysis revealed the fibronectin expression was declined but the β-catenin mRNA levels was unchanged (Figure 4G). Conversely, the protein expressions of β-catenin and fibronectin were elevated in HG-stimulated RTECs transfected with the DNMT3B-OE plasmid (Figure 4H), with only Fibronectin mRNA showing significant upregulation (Figure 4I). Notably, the overexpression of DNMT3B suppressed the expression of SFRP5 compared to HG group (Figure 4J), suggesting that DNMT3B may be the key factor responsible for the methylation of the *sfrp5* gene promoter. Therefore, we proposed that DNMT3B might alleviate the expression of SFRP5 while aggravate the activation of Wnt/β-catenin pathway and the deposition of ECM under high glucose conditions.

### The demethylation drug 5-Aza reversed hyperglycaemic effects in high-glucose stimulated RTECs

**Fig. 5:**
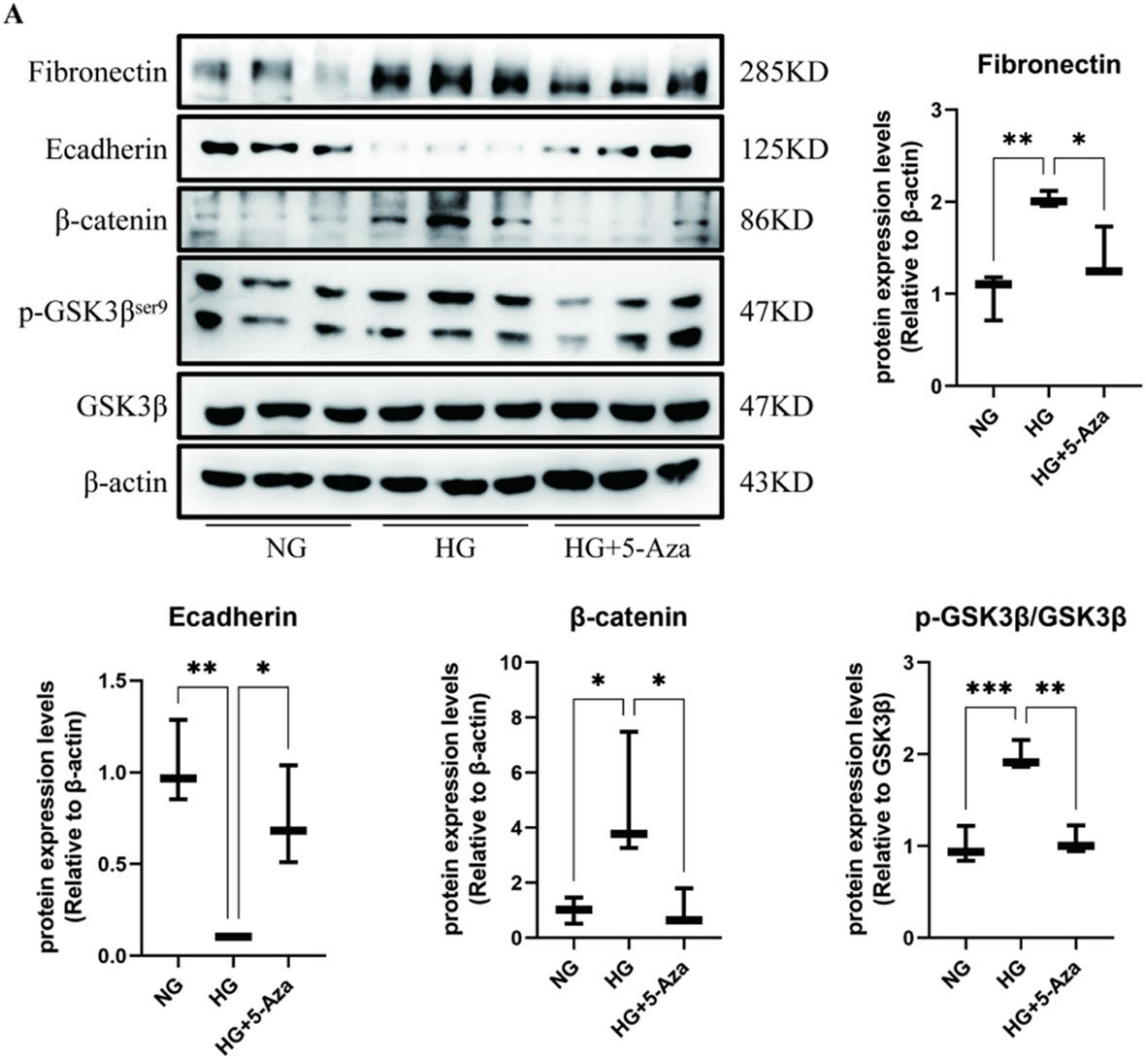
The demethylation drug 5-Aza reversed the hyperglycaemic effects in high-glucose stimulated RTECs. (**A**) The protein expression of ECM-related markers (E-cadherin and fibronectin) was assessed via Western blotting using RTECs after stimulation with or without a DNMT inhibitor (5-Aza, 80 µM) for 48 h (*n* = 3). The data are expressed as means ± SDs. *p < 0.05, **p < 0.01, ***p < 0.001, and ****p < 0.000 vs. control.

We assessed the effect of DNA methylation on the process of DRF. Cells were treated with 5-Aza (80 µM), a DNA methylation inhibitor, accompanied by high-glucose stimulation. 5-Aza treatment inhibited the high glucose-induced upregulation of the Wnt/β-catenin pathway components (Fig. 5A). In addition, 5-Aza attenuated the high glucose-induced suppression of E-cadherin expression and reversed the induction of fibronectin expression (Fig. 5A). The results suggested that aberrant DNA methylation is involved in high glucose-induced Wnt/β-catenin pathway activation and excessive ECM deposition.

## Discussion

The Wnt/β-catenin pathway has been shown to play a significant role in the pathogenesis of renal fibrosis, which has been confirmed by numerous studies in recent years^28–30^. With extensive research on diabetic renal fibrosis, the role of gene methylation in influencing fibrosis progression has gradually been recognized^31,32^. We found that the expression of DNMTs increased to varying degrees in high glucose-stimulated RTECs. Among them, DNMT3B was expressed the most significantly. DNMT3B inhibited the transcription of SFRP5 through the regulation of *sfrp5* promoter methylation, thereby activating the Wnt/β-catenin signalling pathway and exacerbating renal fibrosis in DKD. Thus, our study demonstrated that the aberrant expression of DNMT3B induced by high glucose and the resulting suppression of SFRP5 contribute to renal fibrosis, revealing a novel role of DNMT3B in the pathogenesis of DKD.

The Wnt/β-catenin signalling pathway is evolutionarily conserved and is involved in various critical cellular pathological processes and plays important roles in disease processes such as tumorigenesis^33^fibrosis^34^inflammation^35^ and tissue repairment^36^. Recently, the fibrotic function of Wnt/β-catenin has been reported in different organs, such as the skin^37^ lung^38^ liver^39^ heart^40^ and arteries^41^. Chen et al. reported that Wnt signalling was activated by high glucose in both streptozotocin-induced DKD and *db/db* DKD mice and promoted excessive accumulation of ECM deposition, leading to renal fibrosis^42^. Another study showed that the β-catenin signalling pathway was activated in high glucose-stimulated HK-2 cells^43^. These studies suggest that the Wnt/β-catenin pathway plays a vital role in diabetic kidney fibrosis. In this study, we found that the Wnt/β-catenin pathway was significantly activated in both *db/db* mouse kidneys and high glucose-stimulaed RTECs. This finding is consistent with what previous articles have reported. Our subsequent experiments focused on the upstream regulatory factors of the Wnt/β-catenin signalling pathway.

SFRP family members are important extracellular signalling ligands and antagonists of the Wnt pathway. SFRPs are a group of secreted glycoproteins and include SFRP1, SFRP2, SFRP3, SFRP4, and SFRP5. The expression of SFRPs presents tissue specificity. A recent study revealed that SFRP2 is highly expressed in the urinary bladder, gallbladder, fat tissue and oesophagus but not in the heart or kidney and that SFRP4 is related mainly to female reproduction and development, whereas SFRP1 and SFRP5 are ubiquitously expressed in multiple organs and tissues, including kidneys^44^. SFRP1 is required for the inhibition of renal damage through the noncanonical Wnt/PCP pathway but not the canonical Wnt/β-catenin signalling pathway^45^. Another report suggested that SFRP5 can reduce EMT and slow the course of fibrosis in the renal interstitium by inhibiting the abnormal activation of the Wnt/β-catenin pathway^13^. Consistent with previous research, we observed that the expression of SFRP5 was downregulated in different DKD models and that the Wnt/β-catenin pathway was inactivated after SFRP5 was overexpressed.

Recently, increasing evidences have revealed that DNA methylation is the main mechanism responsible for the silencing of SFRP5. DNA methylation is a crucial universal epigenetic modification. It is a major regulator of transcription and is associated with the silencing of gene expression and determines the expression and function of genes involved in different physiological and pathological processes. Hypomethylation is quite common among global DNA modifications, and abnormal DNA methylation is linked with the development of a variety of human diseases, including cancer^46^neurodegenerative disease^47^ and ageing^48^. There is increasing evidence that DNA methylation of multiple renal fibrosis genes is associated with the pathogenesis of renal fibrosis and promotes the progression of CKD^49,50^. The DNA methylation level in DKD patients has been shown to be markedly different from that in control individuals^51^. On the basis of the above evidence, we propose that SFRP5 may be the key factor regulated by DNA methylation in the progression of DRF. We examined the promoter methylation frequency of *sfrp5* via bisulfite sequencing in DKD models in vivo and in vitro. We found that the frequency of several CpG sites in the *sfrp5* promoter region was increased in the DKD renal tissue and high glucose-stimulated RTECs. These data suggest that a reduction in SFRP5 expression may contribute to the high frequency of methylation in the DNA promoter region of *sfrp5* in DKD.

Gene methylation is catalysed by DNMTs and mediates the inactivated transcription of critical genes that are closely related to the development and progression of renal fibrosis^22^. In mammals, three major DNA methyltransferases, DNMT1, DNMT3A and DNMT3B, have been identified. DNMT1 is responsible for maintaining the DNA methylation status, whereas DNMT3A/3B is responsible for establishing a new DNA methylation pattern. In this study, we explored the expression of DNMTs in high glucose-stimulated RTECs. The results revealed that DNMTs revealed differential expression with DNMT3B demonstrating the highest expression levels. Consistent with our findings, DNMT3B, in contrast to other methyltransferases, has been reported to be preferentially located in CpG islands near the silenced gene promoters and to determine the transcription of these genes^52^. Oba et al. showed that the expression of DNMT1 and DNMT3B, but not that of DNMT3A, was decreased in 15-week-old male *db/db* mice^53^. In addition, Yang et al. reported a time-dependent increase in DNMT3B protein expression in male *db/db* mice^54^. These results indicate that aberrant DNA methylation is closely related to the pathogenesis of DRF that DNMT3B may be emergeing as a major catalytic enzyme of the epigenetic dysregulation. Thus, we hypothesized that DNMT3B plays a major role in gene methylation during the procedure of DRF. This hypothesis is supported by previous research showing that SFRP5 expression is downregulated in allergic rhinitis, that may be attributed to DNMT3B-driven DNA methylation^17^. In our research, shRNA was used to decrease the expression of the DNMT3B gene. Consequently, the reduced protein expression of DNMT3B elevated the SFRP5 levels, thereby suppressing Wnt/β-catenin signalling pathway and attenuating the deposition of ECM. We further used plasmids to overexpress DNMT3B, and the effects were opposite to those observed after silencing the DNMT3B gene. Our results also indicated that the methyltransferase inhibitor 5-Aza reversed the hyperglycaemia-induced Wnt pathway activation and extracellular matrix deposition in RTECs. These data suggest that DNMT3B negatively regulates the expression of SFRP5 in DKD.

While our study focused on DNMT3B’s downstream effects in DKD, the upstream triggers of its overexpression under high glucose remain an unresolved issue. In inherited diseases and cancers, several reports suggest that DNMT3B are regulated at transcriptional and post-translational levels. At the transcriptional level, the promoters of DNMT3B contain multiple binding sites for several transcription factors: the positive factors, such as E2F6^55^, PU.1^56^, and the negative regulators, such as p53^57^, Foxo3a^58^. In addition, at the post-translational modification level, the RNA-binding protein HuR has been demonstrated to regulate DNMT3B by binding to its 3’-UTR and increasing its protein levels in colorectal cancer cells^59^. As Eric et al. researched that the DNMT3B regulation and recruitment on DNA are also regulated by various factors: chromatin remodeling complexes, histone modifications and transcription factors^60^. Future work is needed to dissect these mechanisms, the above regulatory mechanisms in DKD, which might find new therapeutic targets to regulate the epigenetic homeostasis of diabetic kidney diseases.

Taken together, the results of this study confirmed the critical antifibrotic role of SFRP5 in DRF. Moreover, high glucose promoted *sfrp5* promoter hypermethylation in a DNMT3B-dependent manner, activated the Wnt/β-catenin pathway and accelerated the process of renal fibrosis in DKD. This study not only revealed a novel renal fibrosis mechanism but also identified a new target for the treatment of CKD. On the basis of the current results, therapeutic strategies could involve downregulating the expression of DNMT3B. However, further studies are needed to fully elucidate how auxiliary factors are involved in the DNMT3B-mediated epigenetic regulation of SFRP5.

## Materials and methods

### Animals

Twelve specific pathogen-free C57BL/6J mice (8 weeks old, 20 ± 3 g) were purchased from Beijing SIBF Biotechnology Co., Ltd. (Beijing, China). Each subject was individually housed under SPF conditions at a temperature of 23 ± 1 °C with a 12-h light/dark cycle. They had ad libitum access to food and water throughout the feeding experiment. After one week of adaptive feeding at the animal research centre of Guizhou Medical University, the mice were randomly divided into a normal control (NC) group (*n* = 6) and diabetic kidney disease (DKD) (*n* = 6) group using a random number table. The replication of DKD model was induced based on previous studies^61^. In brief, a mouse model of DKD was generated by daily intraperitoneal (ip) injections of streptozocin (STZ; Sigma: S0130.) at 55 mg/kg (dissolved in citrate buffer, pH 4.5) for 5 consecutive days after the mice were fasted for 4 h; an equal dose of solvent was injected into mice in the NC group. Tail vein blood was collected to assess fasting blood glucose (FBG) after 14 days of modelling. The DKD mouse model was considered successfully established when the FBG level in mice was equal to or greater than 16.7 mmol/L; for mice in the NC group, the required FBG level was < 16.7 mmol/L. The mice in each group were provided standard feed and free access to water for 24 weeks. Twenty-four-hour urine volumes were recorded, and 24-h urine samples were collected to quantify 24-h albuminuria before the mice were euthanized by cervical dislocation. Kidneys and blood serum were collected when the animals were killed. Part of the kidney was fixed with 4% paraformaldehyde solution, and another part was stored at − 80 °C for further analysis. The study was conducted with the approval of the Ethics Committee of Guizhou Medical University (Guizhou, China). All animal experiments were approved by the Institutional Animal Care and Use Committee of Guizhou Medical University (NO:2000006). All procedures comply with the standards outlined in the ARRIVE guidelines.

### 24-hour albuminuria quantification method

Mice were housed in metabolic cages (two mice per cage) with ad libitum access to standard diet and water. Urine samples were collected hourly over 24 h using 1.5 mL EP tubes. After the 24-hour total urine volume per cage was recorded, individual mouse urinary output was calculated by dividing the total cage volume by the number of mice per cage. Collected urine samples were immediately centrifuged at 1,500 ×g for 10 min in a pre-cooled centrifuge (4 °C) to obtain supernatants. Urinary protein concentration was quantified using the Urinary Protein Quantification assay kit (Nanjing Jiancheng Bioengineering Institute: C035-2-1). The 24-hour albuminuria was determined by multiplying the urinary protein concentration by the total 24-hour urine volume.

### Histology and immunohistochemistry

Mouse tissues were fixed in formalin, embedded in paraffin, cut into 4-µm-thick sections and mounted on glass slides. After deparaffinization with xylene, the sections were rehydrated in a graded alcohol series, washed in tap water and heated in 0.01 M citric acid buffer (pH 6.0) for 10 min in an autoclave. After natural cooling to room temperature, the sections were treated with 3% H_2_O_2_ at room temperature for 30 min, washed with PBS, and blocked with 0.5% Triton X-100 and 5% normal bovine serum in PBS. The morphological changes in the kidney tissues were observed under a light microscope after haematoxylin-eosin (HE), Masson, PAS and PASM staining. For immunohistochemistry, the sections were incubated with the following primary antibodies at 4 °C overnight: anti-DNMT3B (1:1000; Abcam: ab2851), anti-SFRP5 (1:1000; ProteinTech:14283-1-AP), anti-β-catenin (1:500; Bioss: bs-23663R), and anti-fibronectin (1:1000; Abcam: ab2413). Then the sections were incubated with the corresponding biotinylated secondary antibodies for 30 min at room temperature. The DAB method (A: B = 1 ml: 20 µL) was used to detect and visualize the staining.

### Cell culture

Mouse renal tubular epithelial cells (RTECs; GuangZhou Jennio Biotech Co.,Ltd: JNO-M0020) were cultured in low-glucose Dulbecco’s modified Eagle’s medium (DMEM; Gibco) supplemented with 10% foetal bovine serum (FBS), 100 U/mL penicillin and 100 g/mL streptomycin. The cells were incubated in a humidified incubator at 37 °C with 5% CO2. The cells in the normal control group were cultured in low-glucose DMEM containing 5.5 mM glucose. To explore the effect of high glucose on RTECs, 24.5 mM glucose was added to low-glucose media to a final concentration of 30 mM. To investigate the pharmacological disruption of DNA methylation, we treated the cells with 5-Aza-2’-deoxycytidine (5-Aza, 20 µM; MedChemExpress: HY-A0004).

### Transient cell transfection

All transient transfections were performed with LipofectamineTM 2000 Reagent (Invitrogen:11668030). For gain- and loss-of-function studies, DNMT3B-sh plasmid (Lv-shRNA-GP), DNMT3B-OE plasmid (pCMVGFPPuro01-Dnmt3b: NM_001122997) or SFRP5-OE plasmid (pCMVGFPPuro05-Sfrp5:NM_001107591) were purchased from Shanghai Yile Biological Co., Ltd. (Shanghai, China) and transfected into cells following an established transfection protocol. Briefly, exponentially growing RTECs were seeded in 6-well plates at a density of 1 × 10^6^ cells per well and were grown overnight to a confluence of 50–60%. The plasmids was subsequently added to the culture media at a final concentration of 100 nM according to the manufacturer’s recommendation. After 8 h of transfection, the medium was replaced with high-glucose medium, and the cells were incubated for 48 h. The transfection efficiency (90%) was measured via Western blotting.

### Western blot

Total protein from kidney tissue or RTECs was obtained with a protein extraction kit (Solarbio: EX1102) according to the manufacturer’s instructions. Protein was separated via 10% sodium dodecyl sulfate‒polyacrylamide gel electrophoresis (SDS‒PAGE). Electrophoresis was terminated when the bromophenol blue dye front (containing proteins) had migrated through approximately two-thirds of the gel. Subsequently, the separated proteins were electrophoretically transferred onto polyvinylidene fluoride (PVDF) membranes. The membranes were blocked with 5% (w/v) non-fat dry milk in Tris-buffered saline containing 0.1% Tween-20 (TBST) for 1 h at room temperature. The membranes were blocked with 5% (w/v) skimmed milk in TBST (Tris-buffered saline containing 0.1% Tween 20) for 1 h at room temperature. Following three washes with TBST (5 min per wash), the membranes were trimmed into specific regions based on the molecular weights of the target proteins. These membrane strips were then incubated with corresponding primary antibodies: anti-β-actin (1:5000; ProteinTech:66009-1-Ig), anti-DNMT3B (1:1000; Abcam: ab2851), anti-SFRP5 (1:1000; ProteinTech:14283-1-AP), anti-E-cadherin (1:2000; ProteinTech:20874-1-AP), anti-β-catenin (1:500; Bioss: bs-23663R) and anti-fibronectin (1:1000; Abcam: ab2413) in TBST with gentle agitation at 4 °C overnight for 16 h. Following three washes with TBST (5 min/wash), membranes were incubated with horseradish peroxidase (HRP)-conjugated secondary antibodies (1:5000; ProteinTech: SA00001-2) for 1 h at room temperature. Protein bands were visualized using the enhanced chemiluminescence (ECL) reagent (Smart lifesciences: H31500) and quantified using ImageLab software. Relative protein expression levels were normalized to β-actin through greyscale value analysis.

### RNA extraction and rt‒qpcr

Total RNA was isolated from renal tissue and RTECs using TRIzol (Invitrogen). A RevertAid™ First Strand cDNA Synthesis Kit (Thermo: K16225) was used for reverse transcription of the purified RNA. cDNA was subjected to RT-qPCR with Talent qPCR PreMix (SYBR Green) (Tiangen: FP209). The corresponding sequences of primers used to detect the expression of DNMT3B, SFRP5, β-catenin and fibronectin are shown in Table 1. Fold changes were calculated via relative quantification (2^− Ct^ method).

**Table 1:** The sequences of the primers used for PCR.

Name	Primer sequence
β-actin	Forward: 5′-CGTGCGTGACATCAAAGAGA-3′
	Reverse: 5′-CCAAGAAGGAAGGCTGGAAAA-3′
DNMT3B	Forward: 5′-TGAATGAAGAAGAGGGTGC-3′
	Reverse: 5′-TCCAAGACTGGGGGTGAGG-3′
β-catenin	Forward: 5′-GGCAACCCTGAGGAAGAAGA-3′
	Reverse: 5′-TGCGTGAAGGACTGGGAAAA-3′
Fibronectin	Forward: 5′-GAGGGGAGTGGAAGTGTGAG-3′
	Reverse: 5′-TGAGTCTGCGGTTGGTAAAT-3′
Sfrp5	Forward: 5′-GAAGCTGGTTCTGCACATGA-3′ Reverse: 5′-AGGGAACAGGGGTAGGAGA-3′

### DNA methylation detection via BSP

Genomic DNA was extracted from RTECs or kidney tissues using a genomic DNA kit (Magen: D3018). Bisulfite modification of the DNA was performed with a fast DNA Bisulfite Kit (Qiagen:59826) in accordance with the manufacturer’s protocol. After bisulfite modification, unmethylated cytosine was converted to uracil by bisulfite, while methylated cytosine remained unabridged. BSP was performed using SFRP5 methylation-specific primers (Table 2). The PCR products were subjected to agarose gel electrophoresis in a 2% Tris/borate/EDTA (TBE) agarose gel. The recycling process was carried out according to the specifications of an agarose gel DNA recovery kit (Tiangen: DP219). Reclaimed DNA was ligated into the PGM-T vector according to the manufacturer’s instructions (Tiangen: VT202) and then transformed into DH5α competent cells (Tiangen: CB101) using a pMD^TM^19-T vector cloning kit (Takara:6013). The DNA was cultured in Luria-Bertani (LB) solid medium supplemented with 20 mg/ml X-gar, 40 mg/ml isopropyl-b-D-thiogalactopyranoside (IPTG) and 100 mg/ml ampicillin in a constant-temperature shaker at 37 °C. Then, at least ten single colonies (white clones) were selected. The corresponding plasmid DNA was extracted after bacterial culture amplification, after which the methylation status of each site was assessed.

**Table 2:** The sequences of the SFRP5 primers used for the BSP analysis.

Name	Primer sequence
*Sfrp5*	Forward: 5’ GGAAATTAGAGTTGAGTAGGGA 3’
	Reverse: 5’ AAAAACTCAATTTACCAACCAT 3’

### Statistical analysis

All the statistical analyses were performed via SPSS 22.0 software, and differences with *P* < 0.05 were considered statistically significant. The data are representative of at least three independent experiments and are presented as means ± SDs. Independent-sample t tests were used to analyse differences between two groups, and one-way ANOVA was used to compare multiple groups followed by Tukey’s HSD post-hoc test.

## Electronic supplementary material

Below is the link to the electronic supplementary material.

Supplementary Material 1

## Data Availability

The datasets produced or analyzed in this study are accessible from the corresponding author upon reasonable request.
