# Supplementary material for: DNMT3B aggravated renal fibrosis in diabetic kidney disease via activating Wnt/β-catenin signaling pathway
Source: Sci Rep. 2025 Jul 1;15:21070. doi: 10.1038/s41598-025-06713-3 (PMC12218845; doi:10.1038/s41598-025-06713-3)
Supplement: Supplementary file 1 — Supplementary Material 1 [file 41598_2025_6713_MOESM1_ESM.docx]

**Actin for Fig 1A**


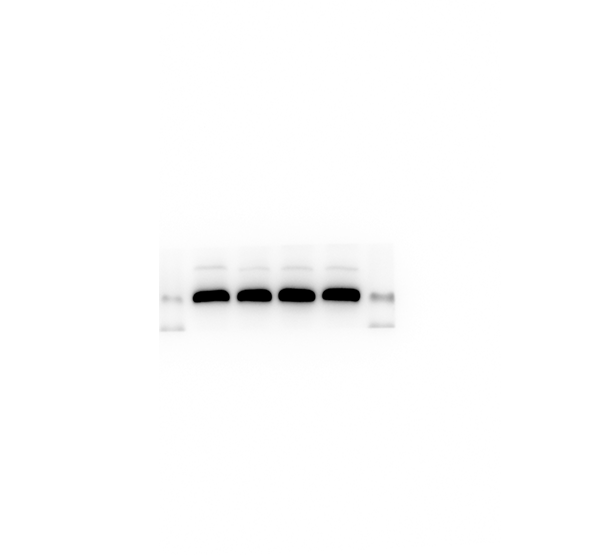


35KD

40KD

**GSK3β for Fig 1A**


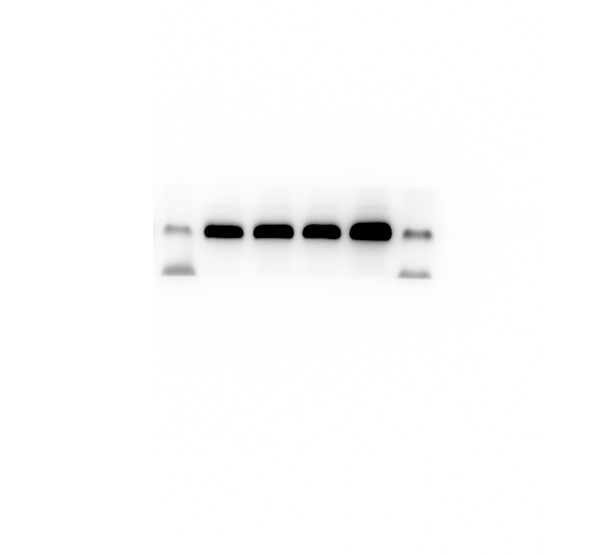


35KD

40KD

**p-GSK3β^ser92^ for Fig 1A**


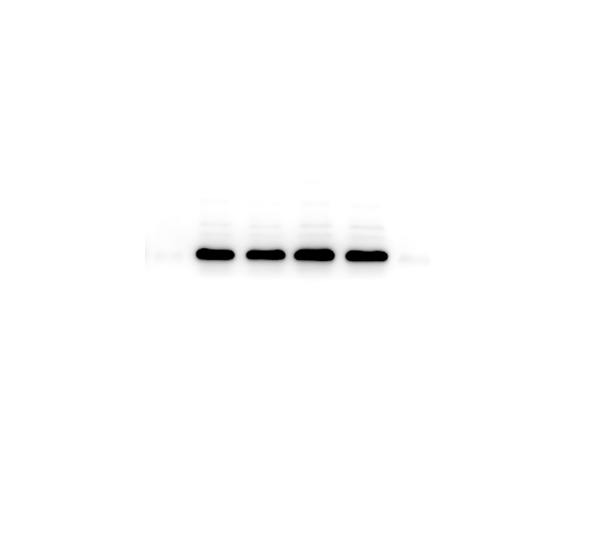


40KD

**β-catenin for Fig 1A**
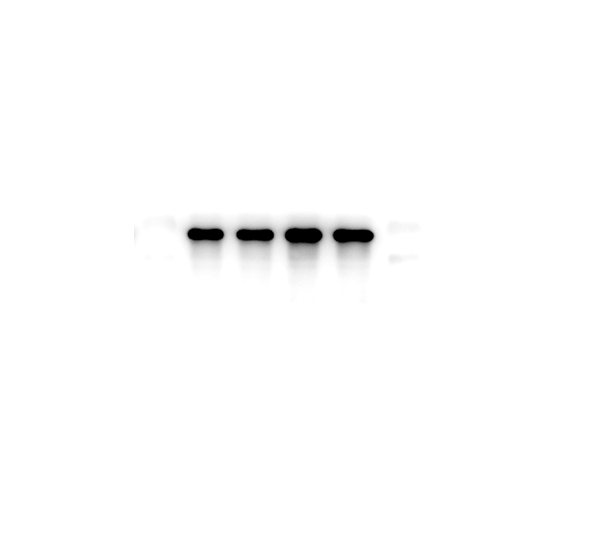


70KD

100KD

**Actin for Fig 1C**


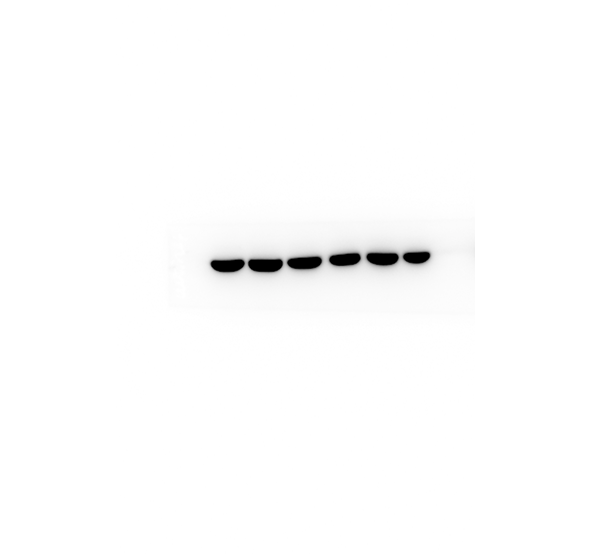


40KD

**β-catenin for Fig 1C**


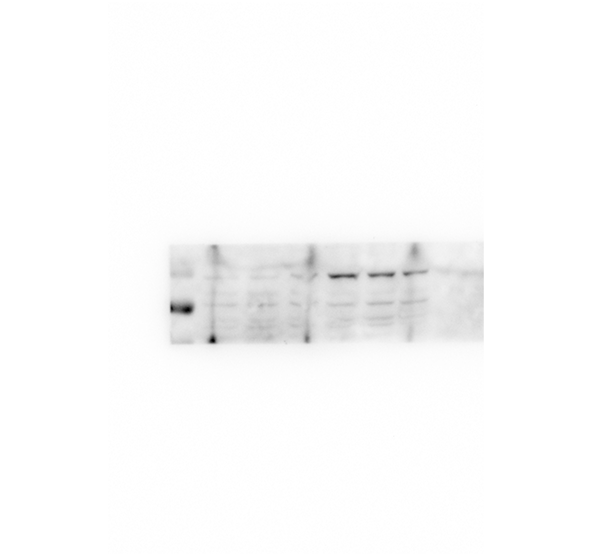


100KD

70KD

**GSK3βfor Fig 1C**


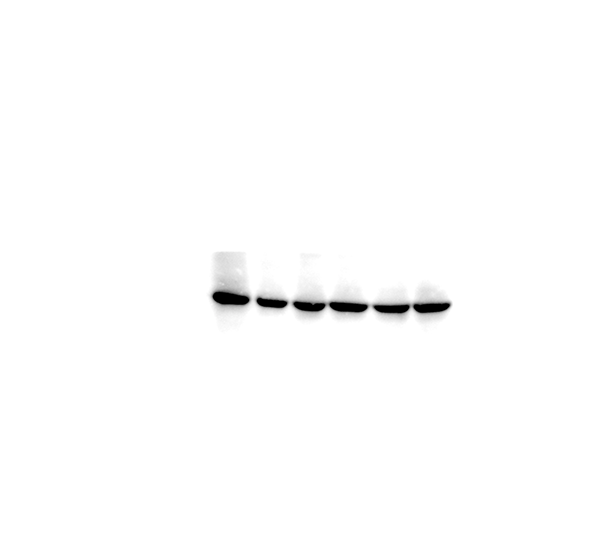


40KD

**p-GSK3β^ser92^ for Fig 1C**


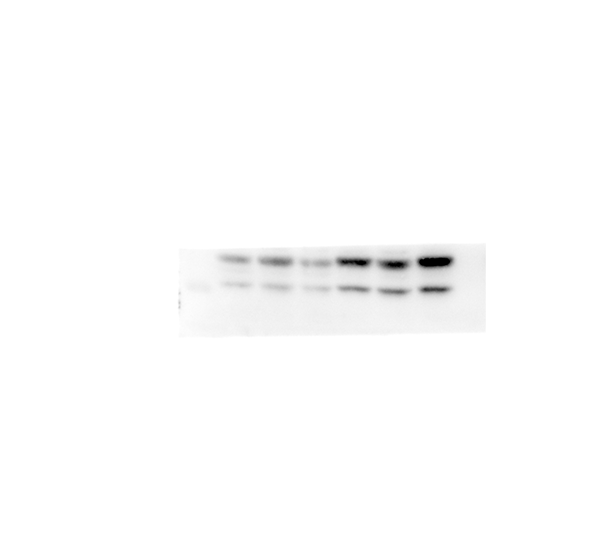


40KD

35KD

**Actin to E-cadherin for Fig 1F**


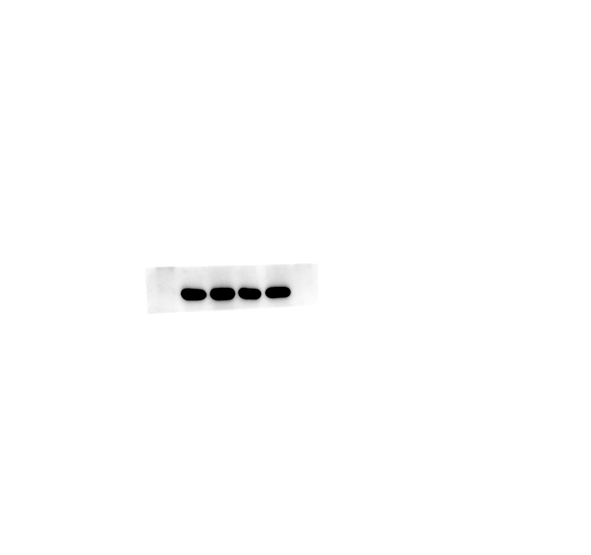


40KD

**Actin to fibronectin for Fig 1F**


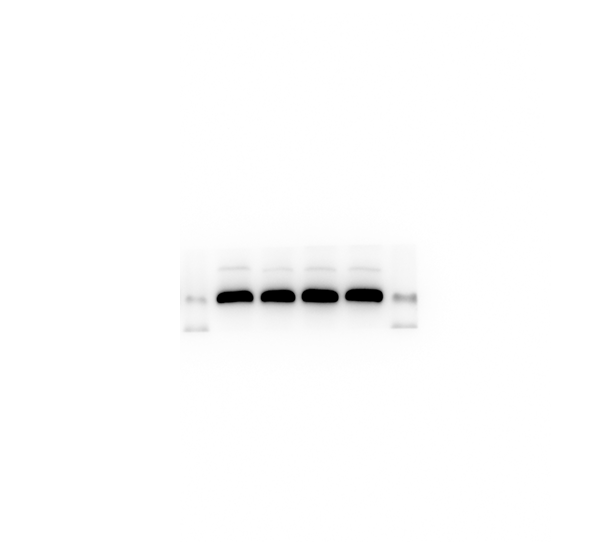


35KD

40KD

**E-cadherin for Fig 1F**
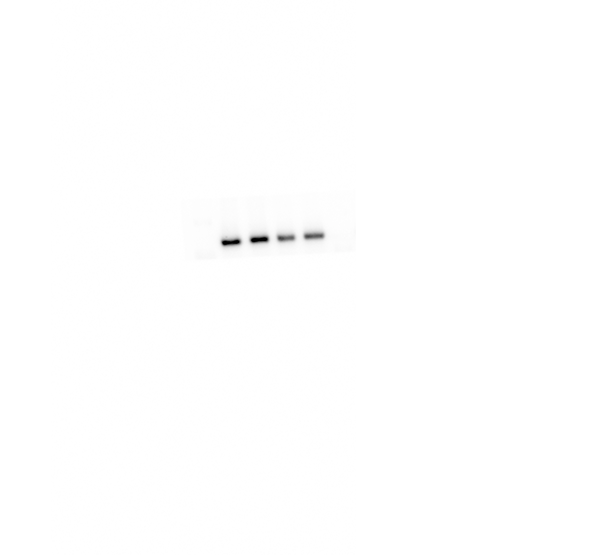


100KD

130KD

**Fibronectin for Fig 1F**


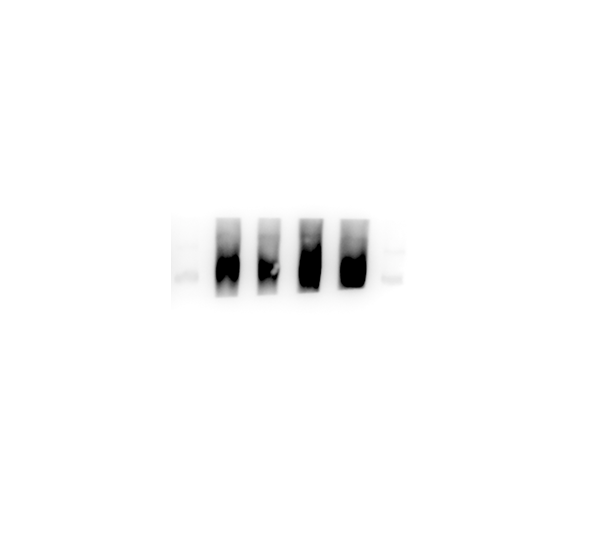


300KD

250KD

**Actin to E-cadherin for Fig 1H**


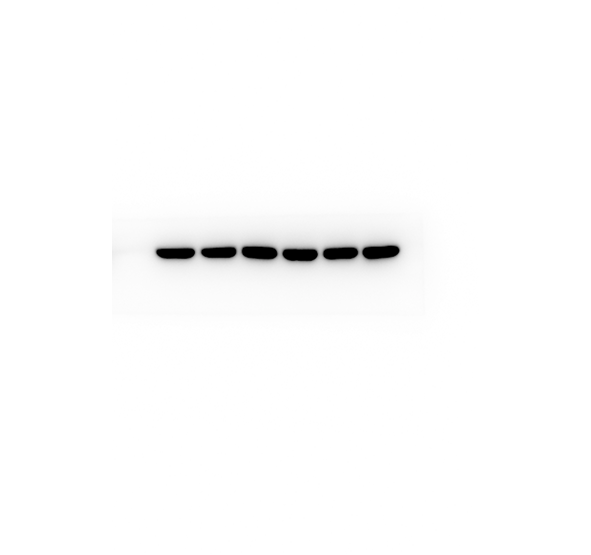


40KD

**Actin to fibronectin for Fig 1H**


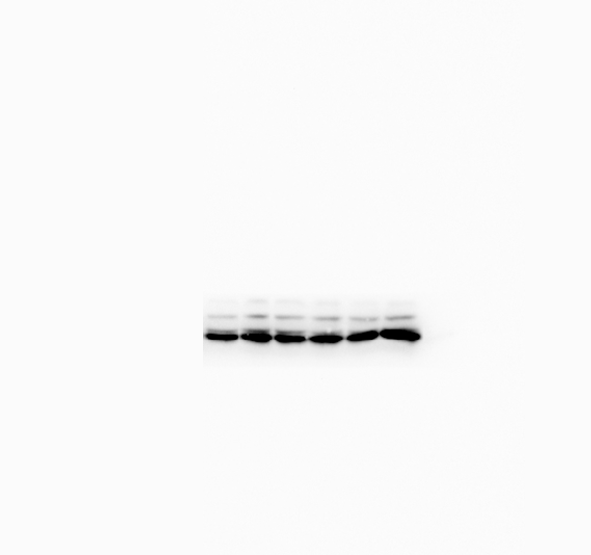


40KD

**E-cadherin for Fig 1H**


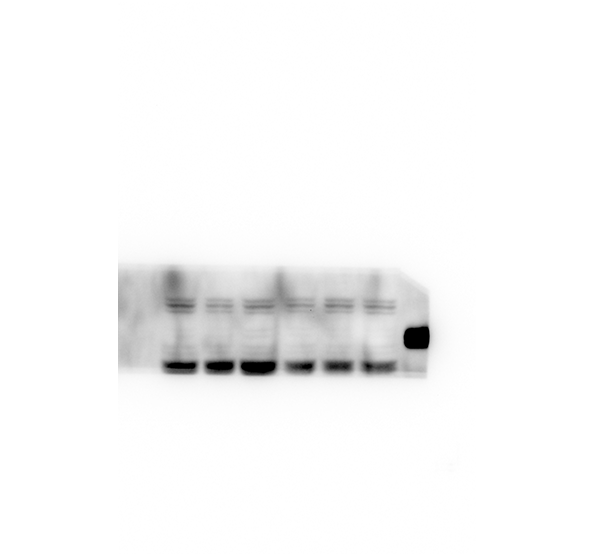


130KD

**Fibronectin for Fig 1H**


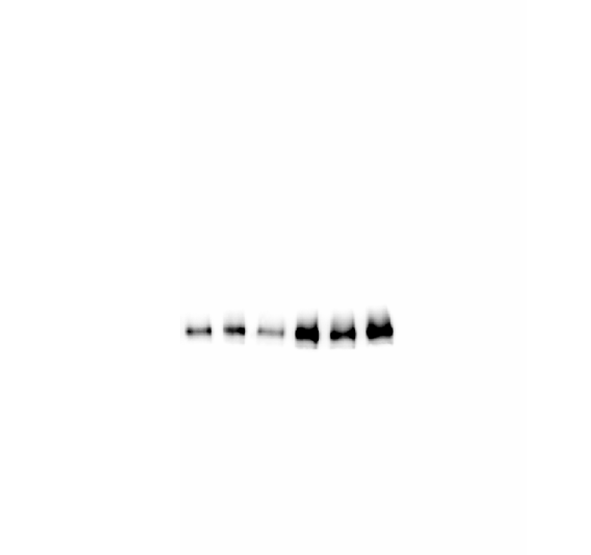


300KD

250KD

**Actin for Fig 2A**


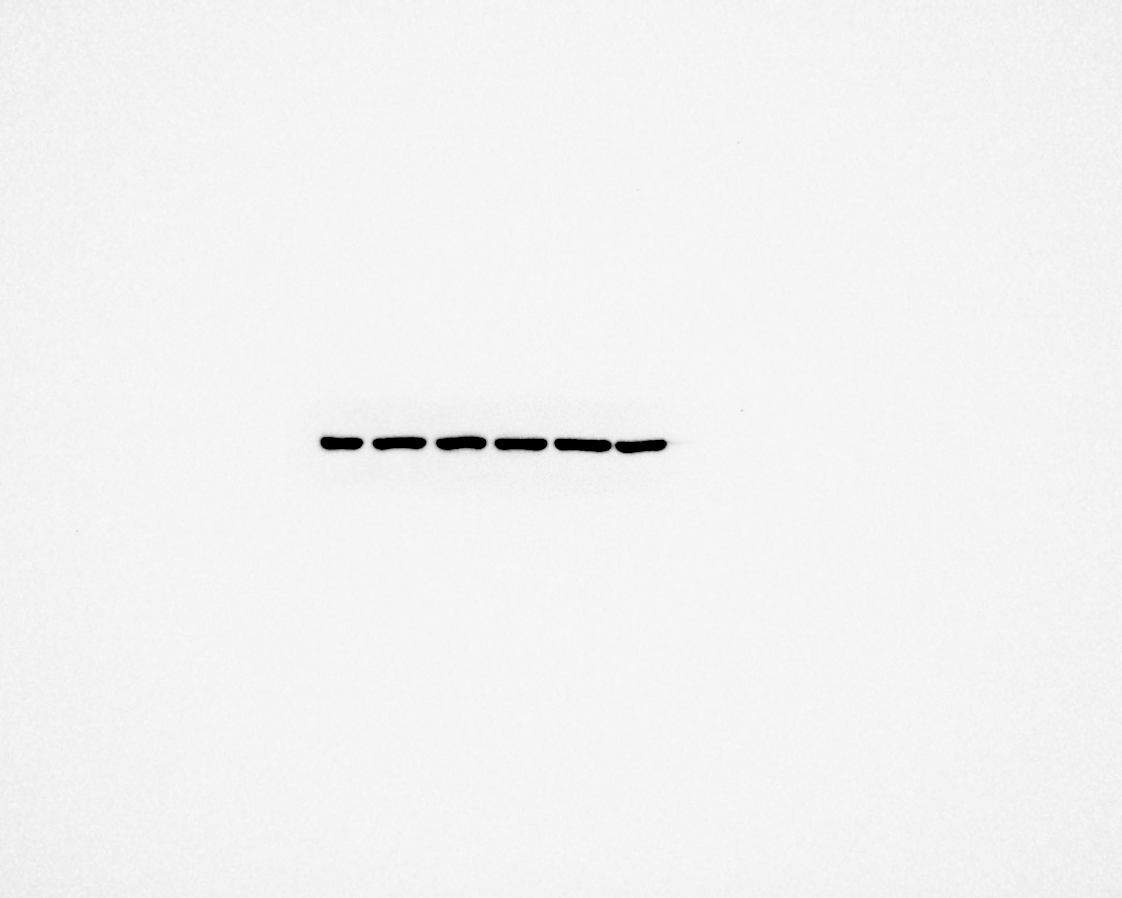


40KD

**sfrp5 for Fig 2A**


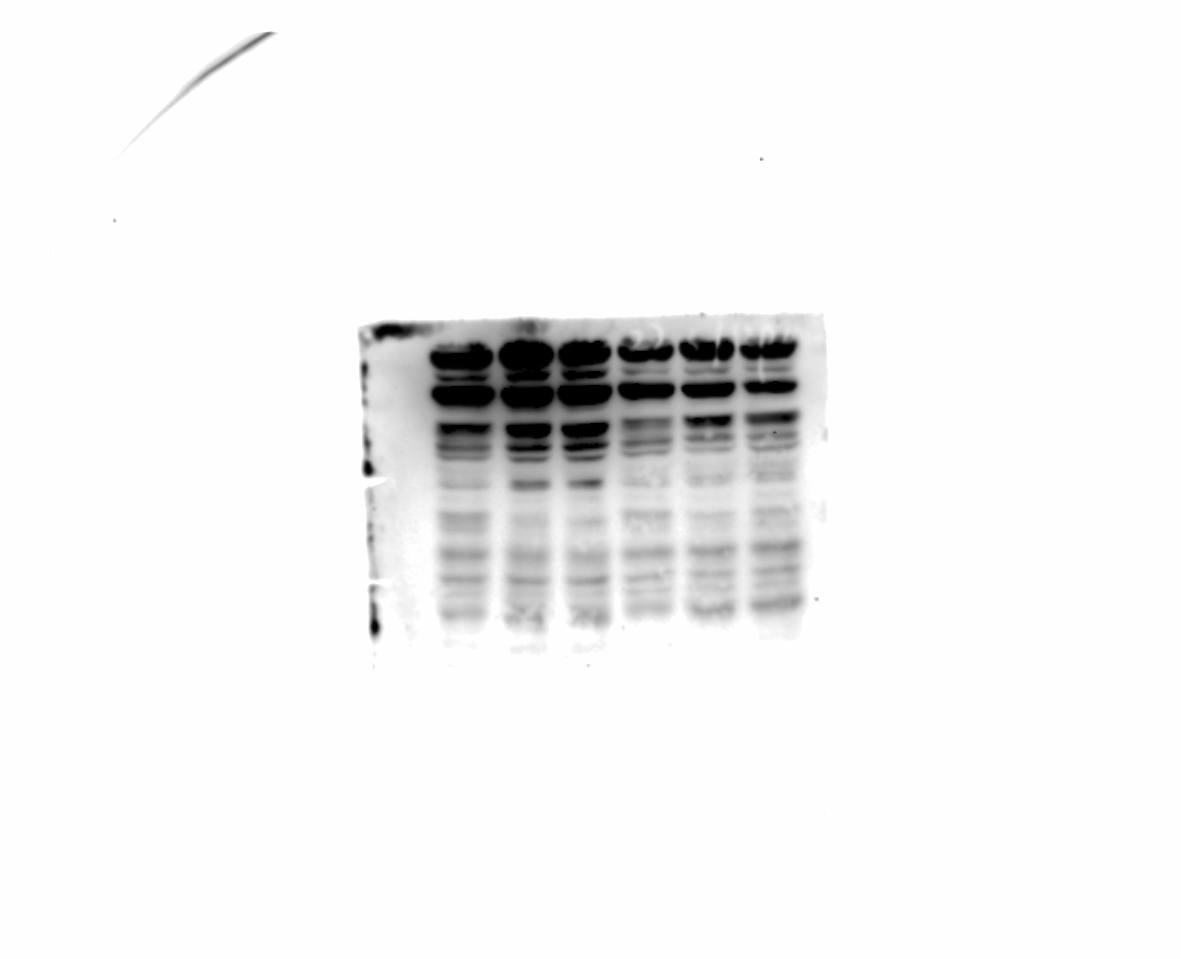


40KD

**Actin for Fig 2B**


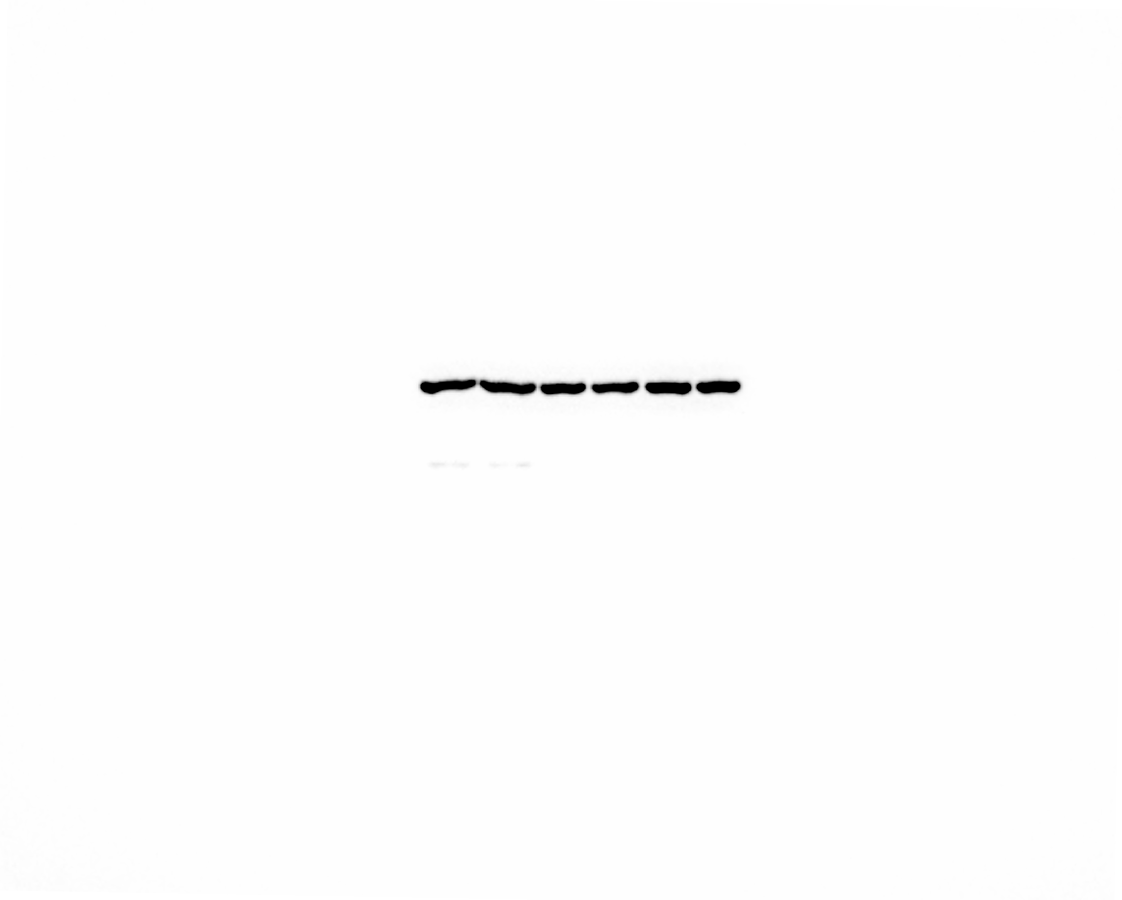


40KD

**sfrp5 for Fig 2B**


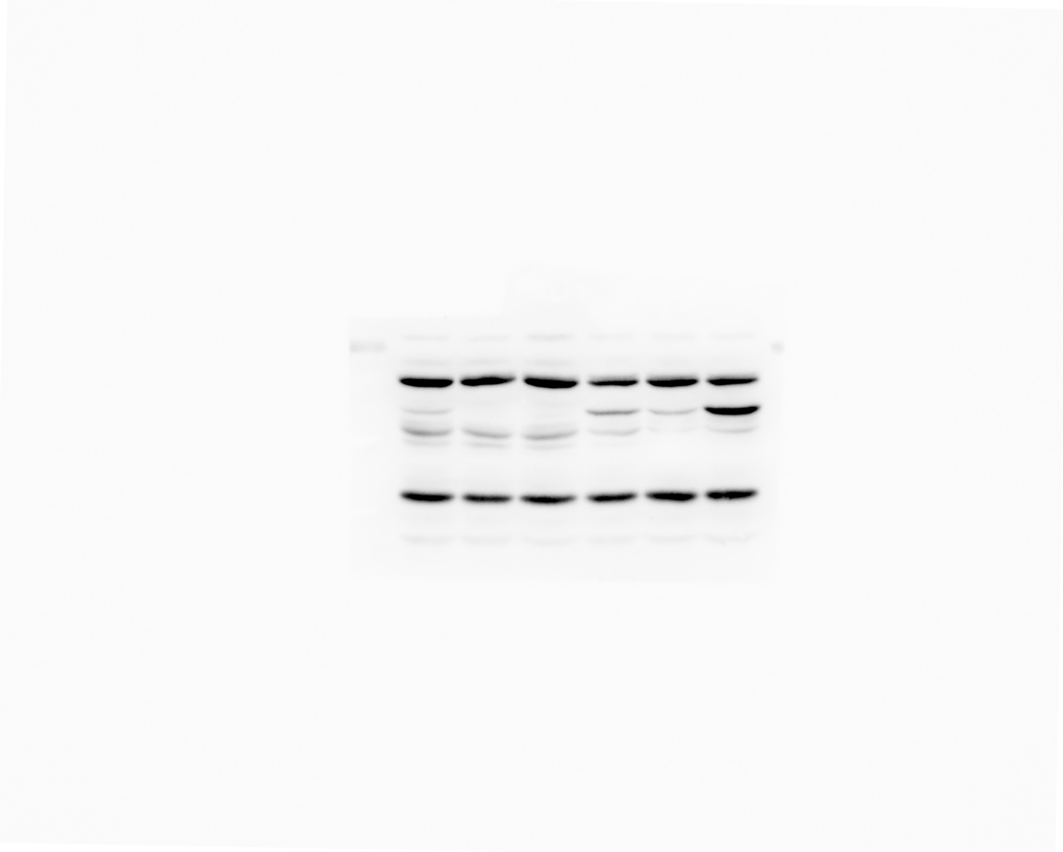


40KD

**Actin for Fig 2E**


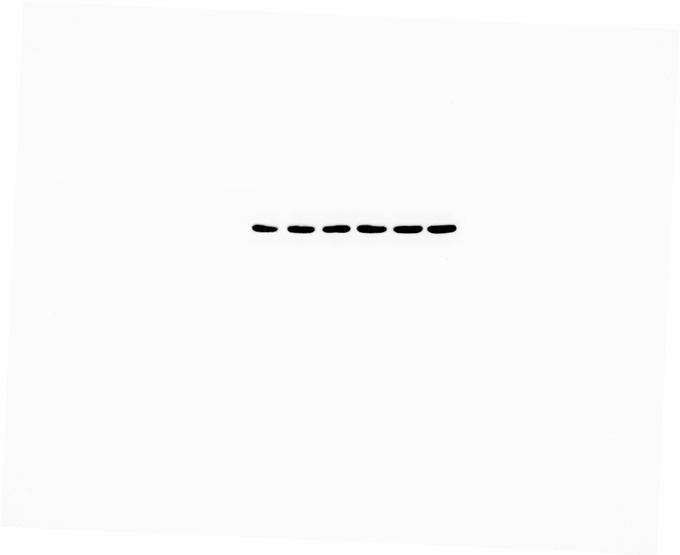


40KD

**E-cadherin for Fig 2E**


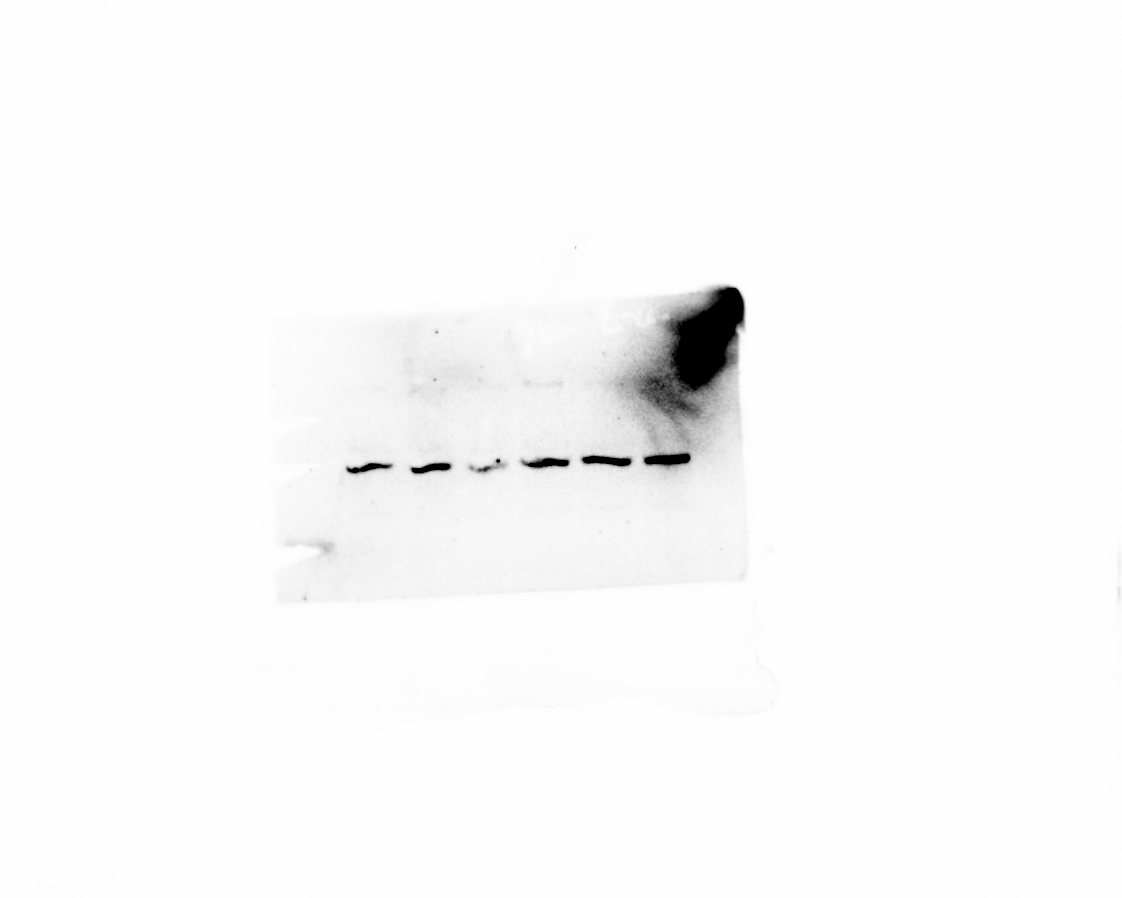


130KD

**GSK3β for Fig 2E**


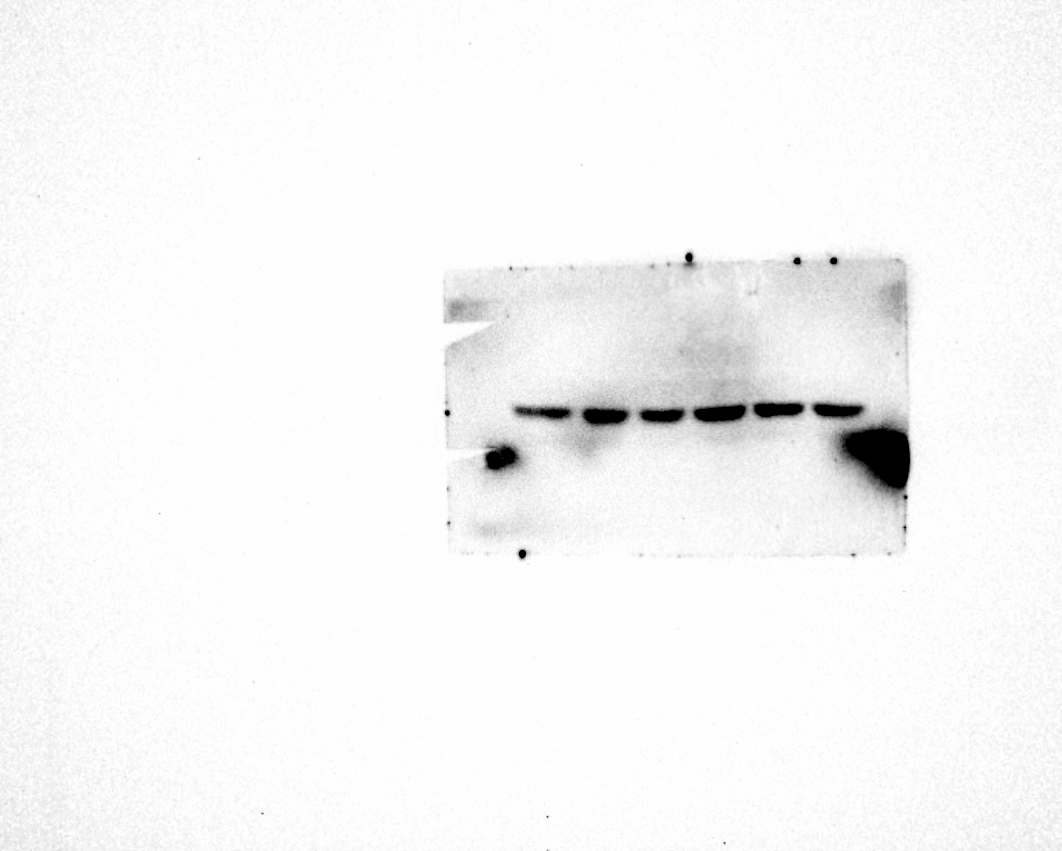


40KD

**p-GSK3β^ser92^ for Fig 2E**


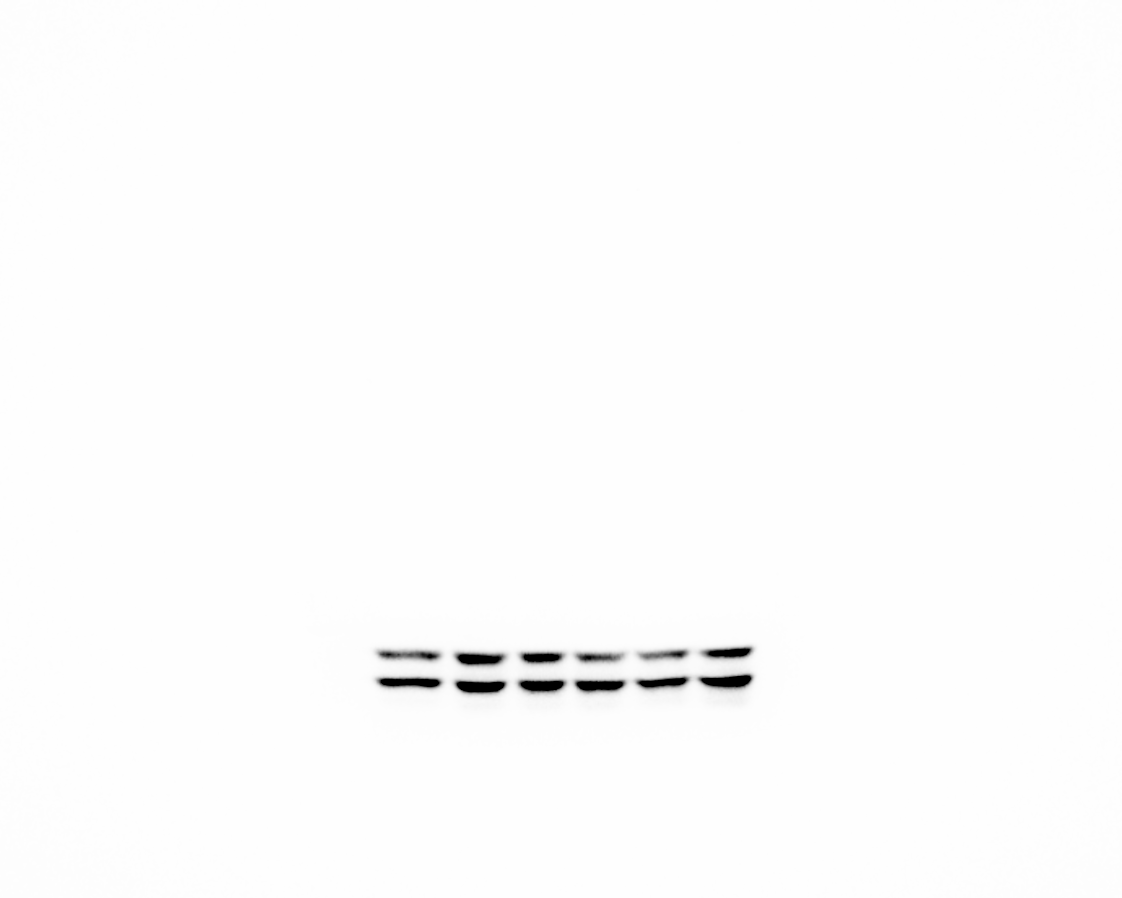


40KD

**β-catenin for Fig 2E**


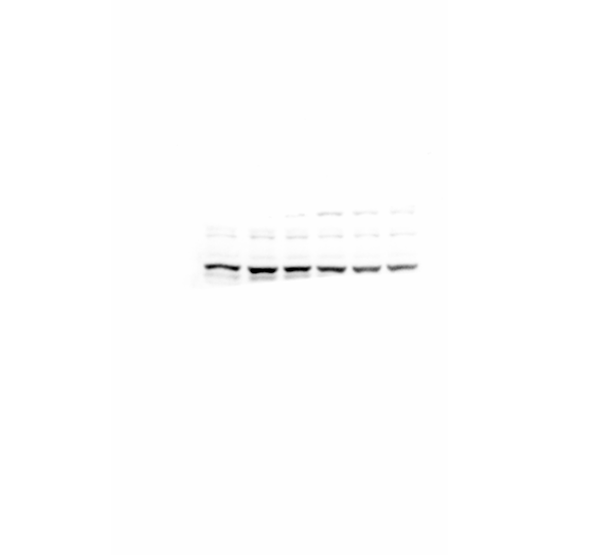


100KD

**Actin for Fig 4A**


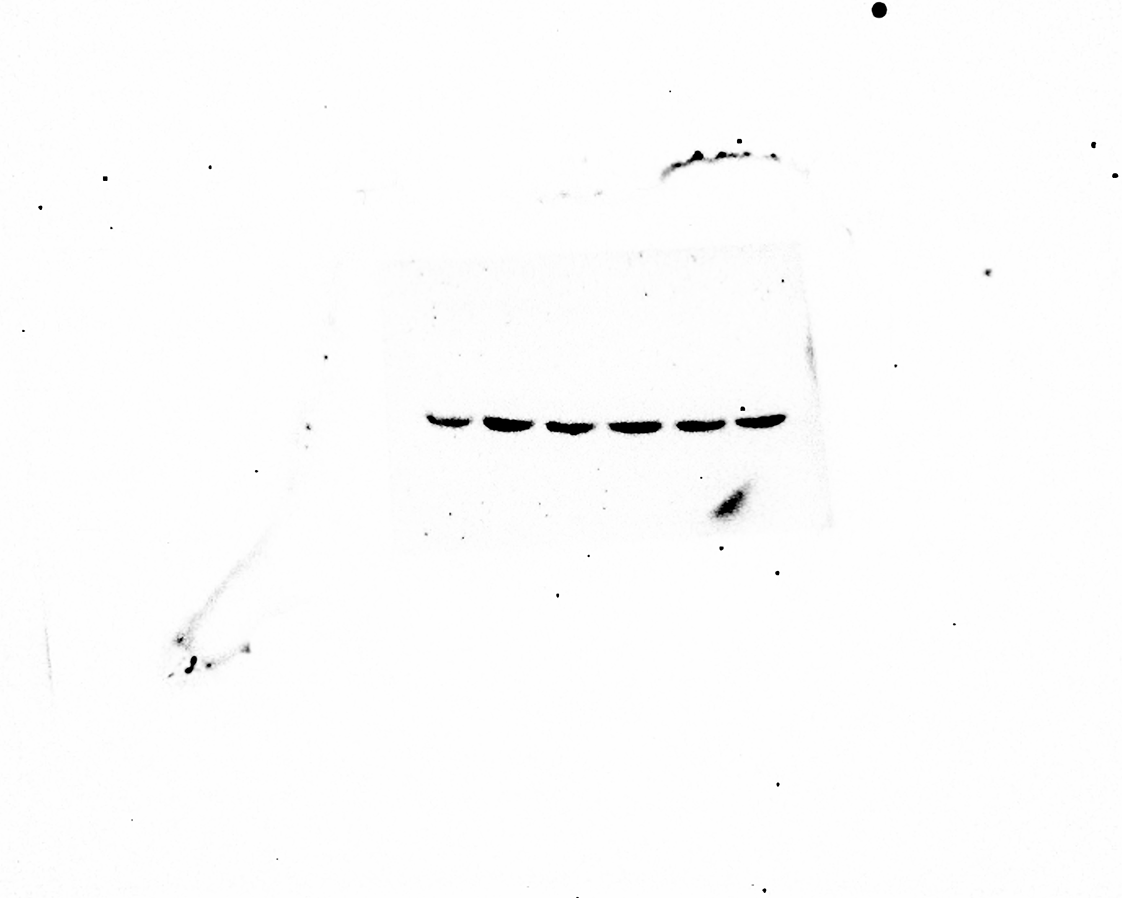


40KD

**dnmt1 for Fig 4A**


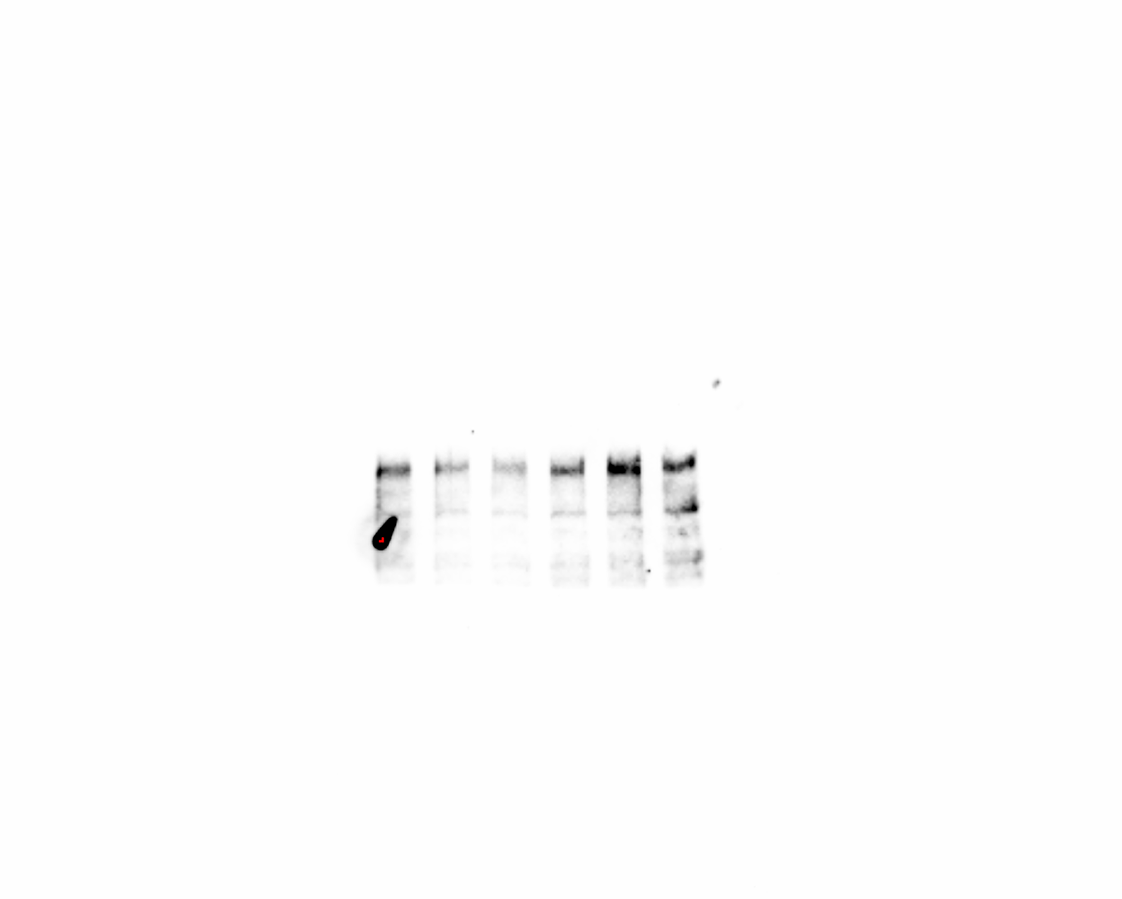


180KD

**dnmt3a for Fig 4A**


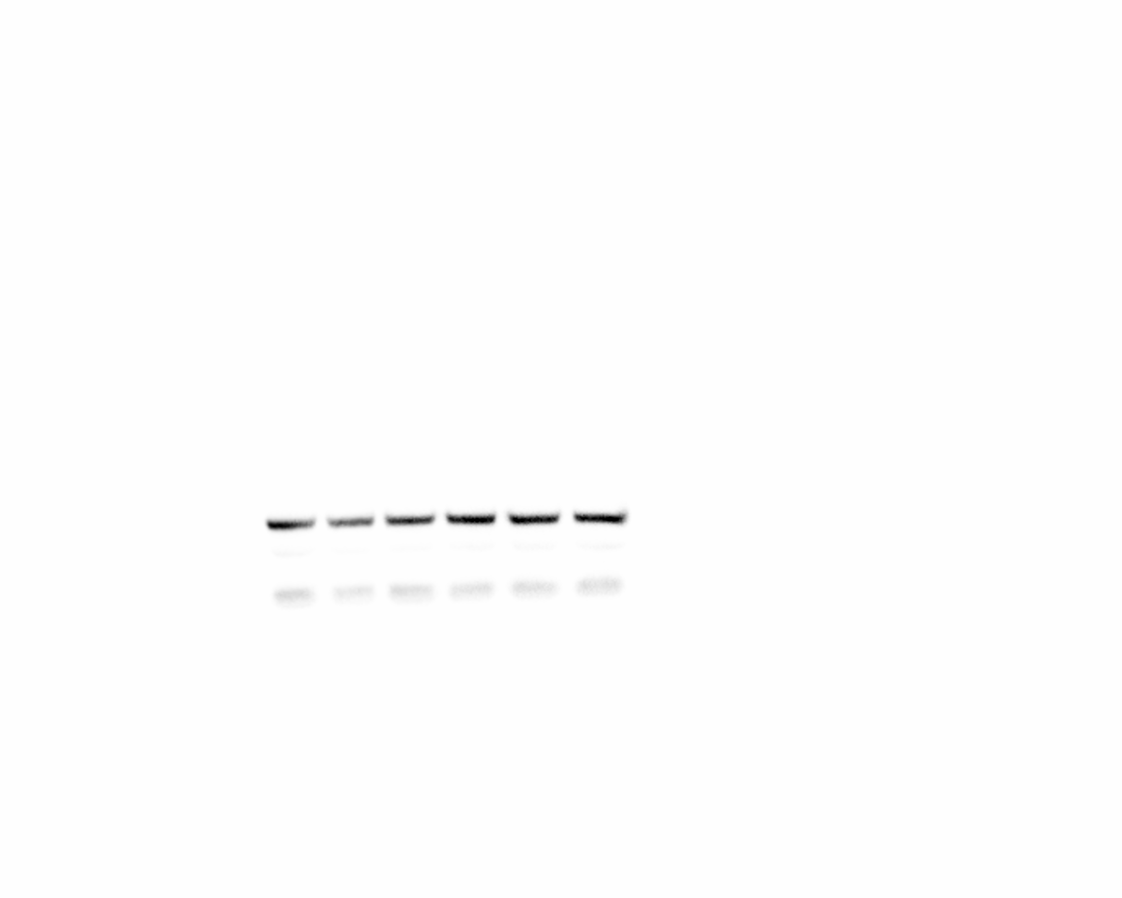


130KD

**DNMT3B for Fig 4A**


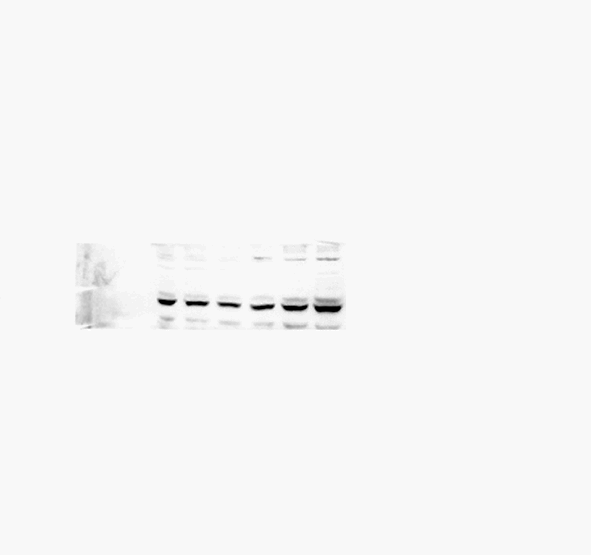


70KD

**dnmt3L for Fig 4A**


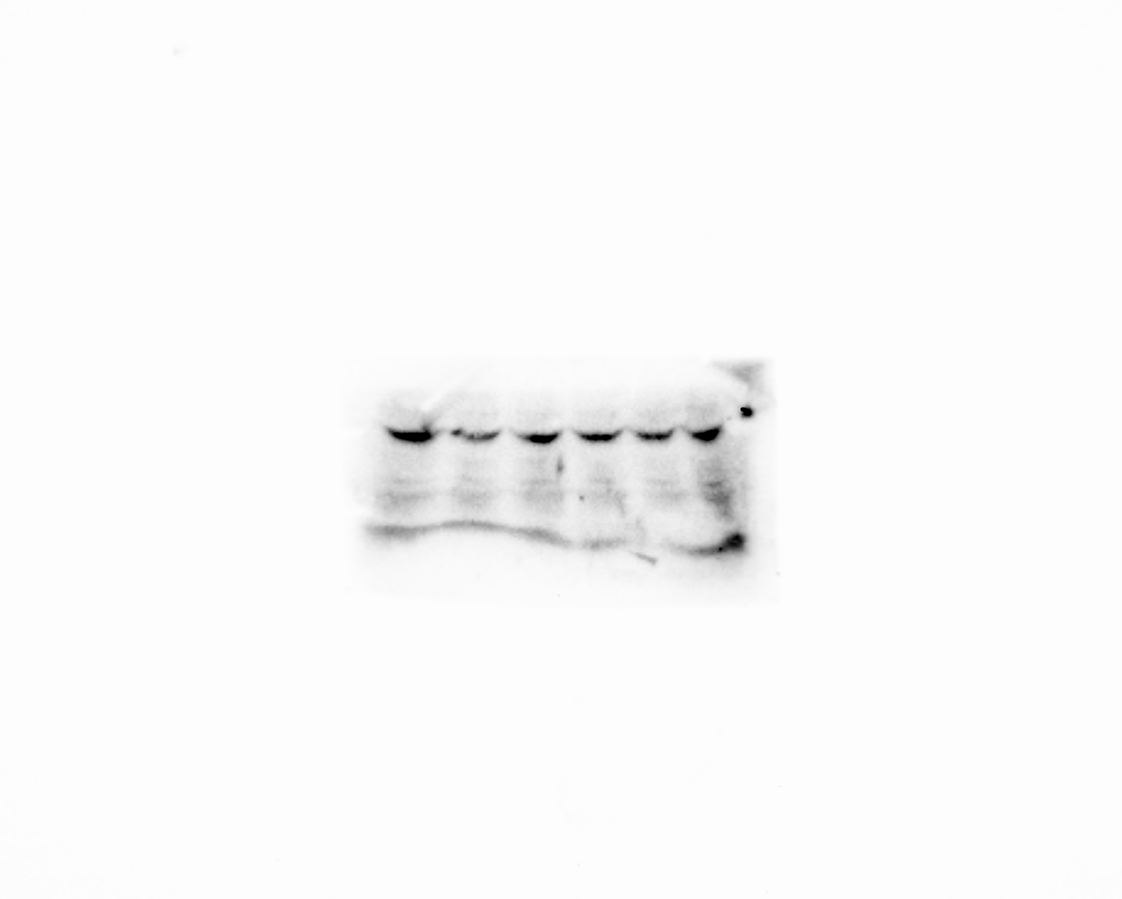


40KD

**dnmt2 for Fig 4A**


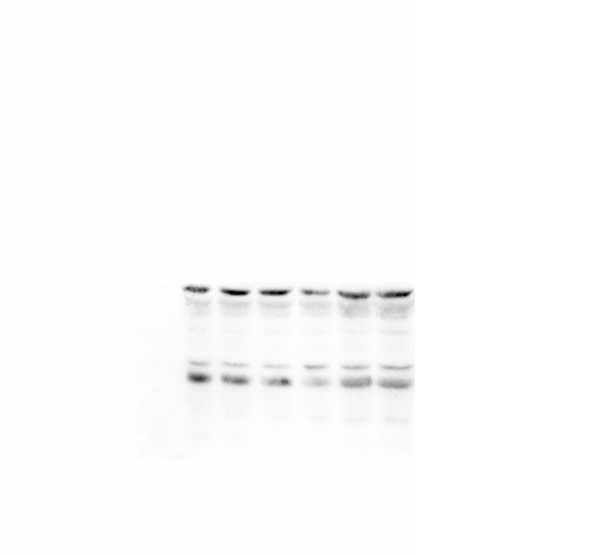


40KD

**Actin for Fig 4B**


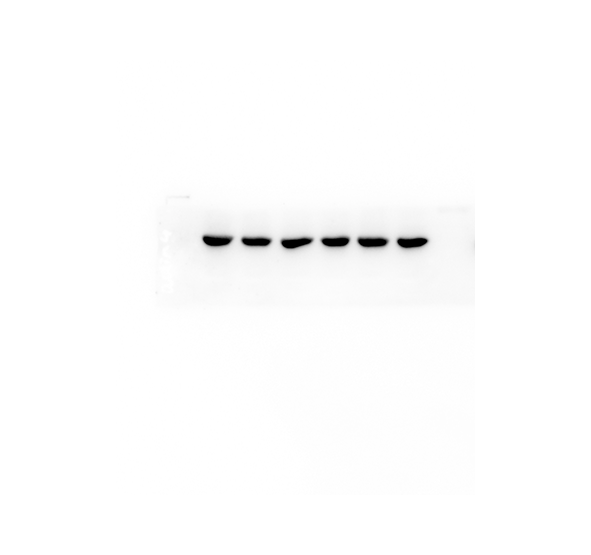


40KD

55KD

**dnmt3b for Fig 4B**


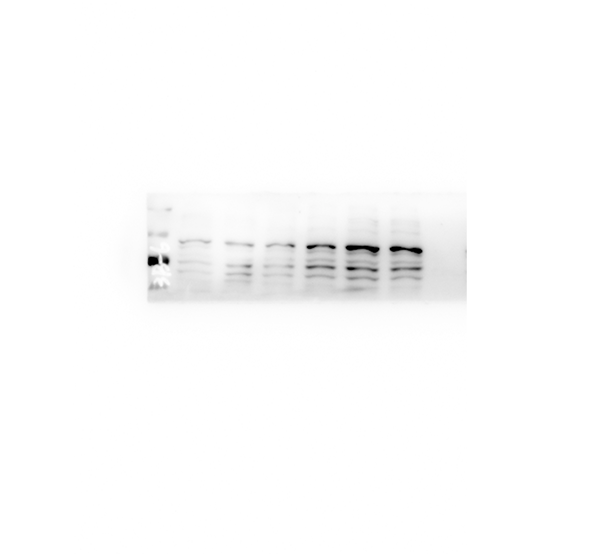


100KD

70KD

**Actin for Fig 4F**


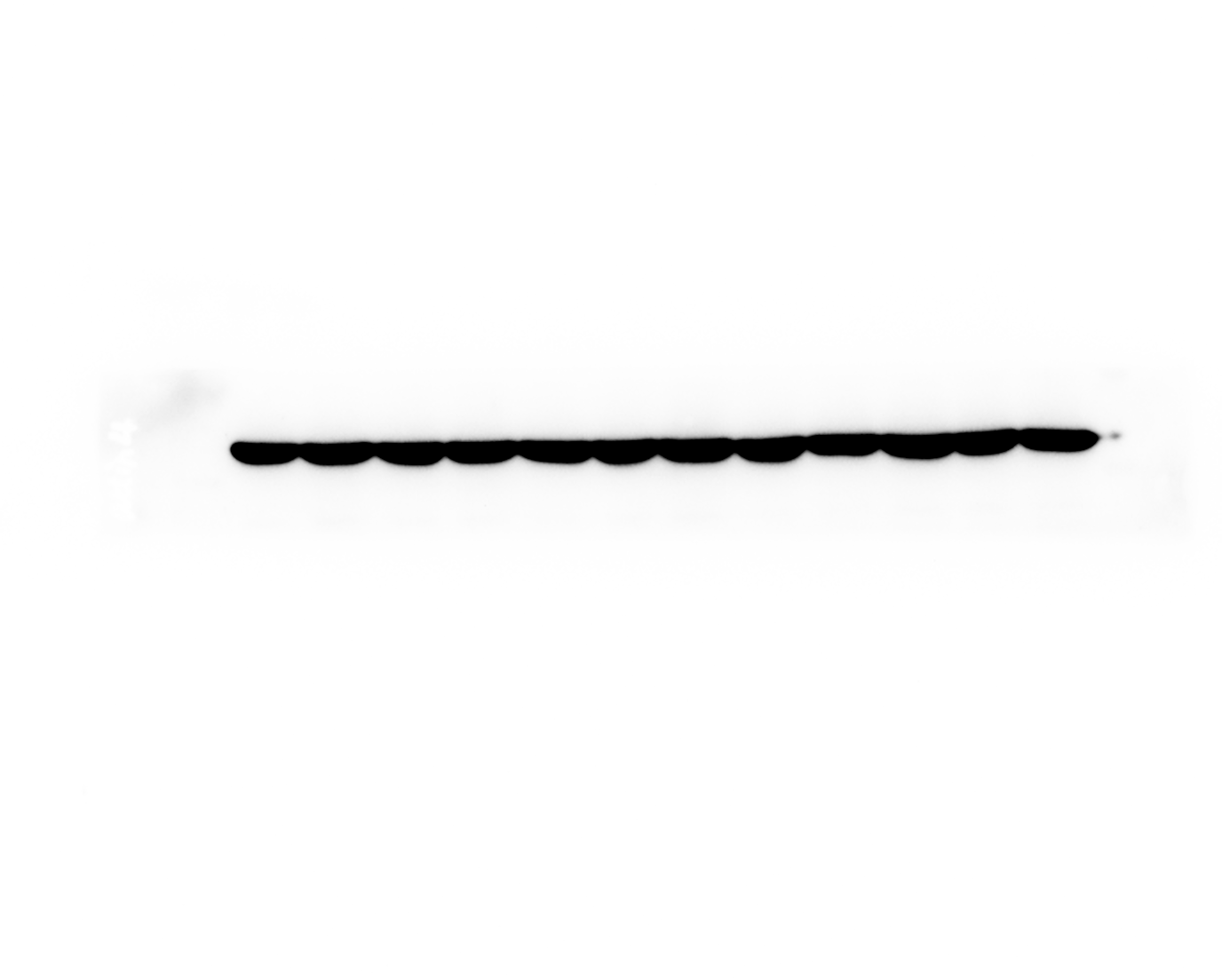


40KD

**E-cadherin for Fig 4F**


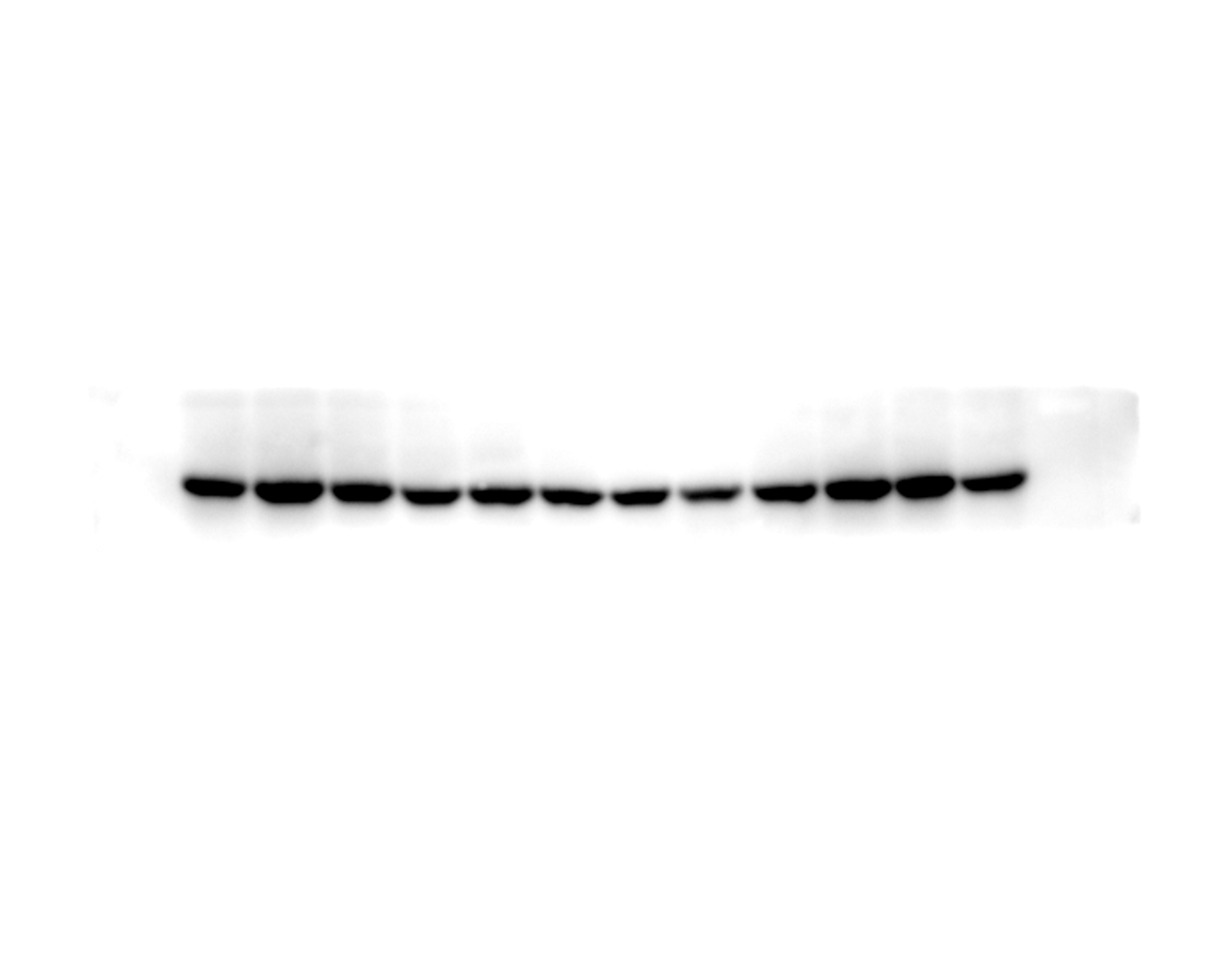


130KD

**Fibronectin for Fig 4F**


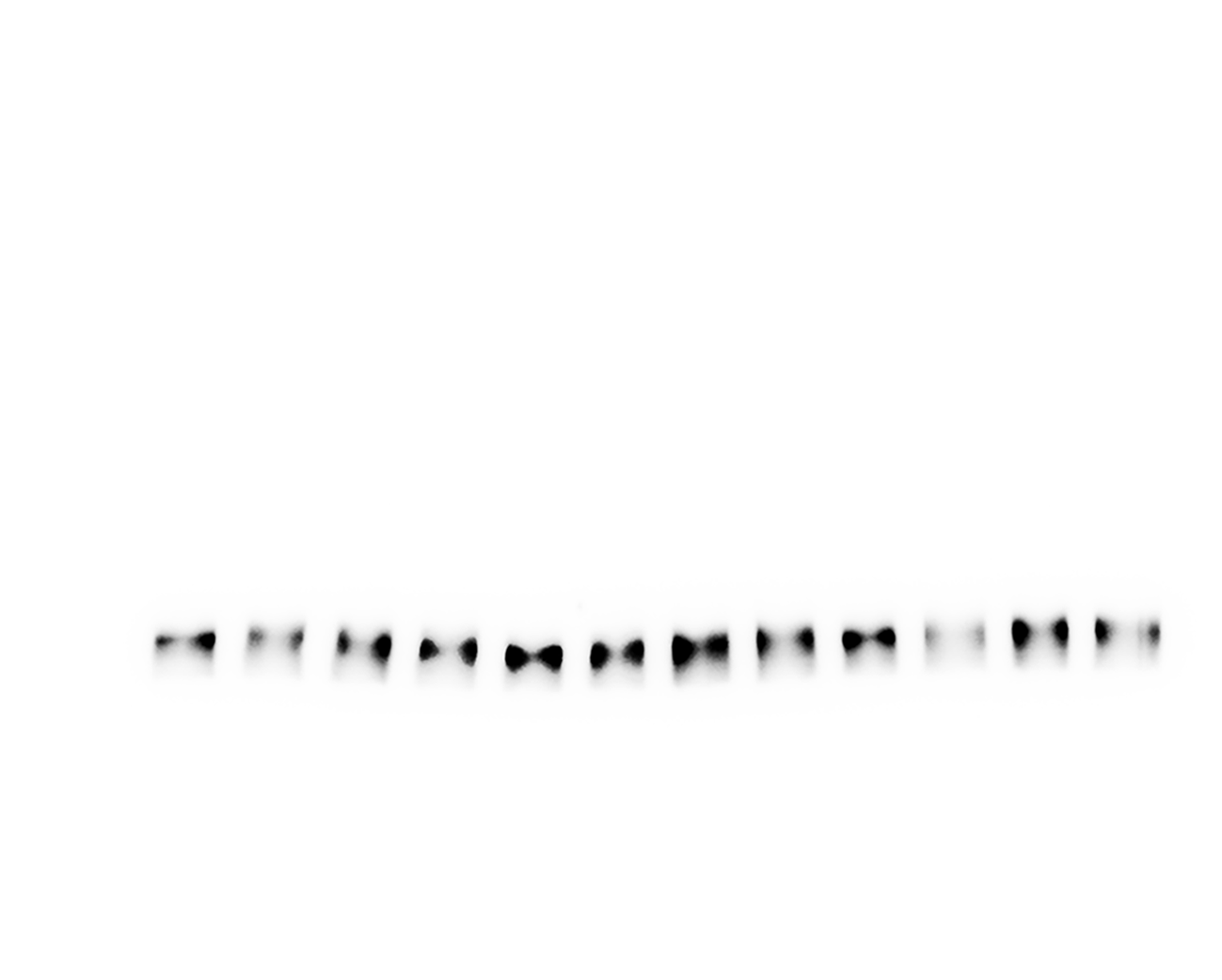


250KD

300KD

**β-catenin** **for Fig 4F**


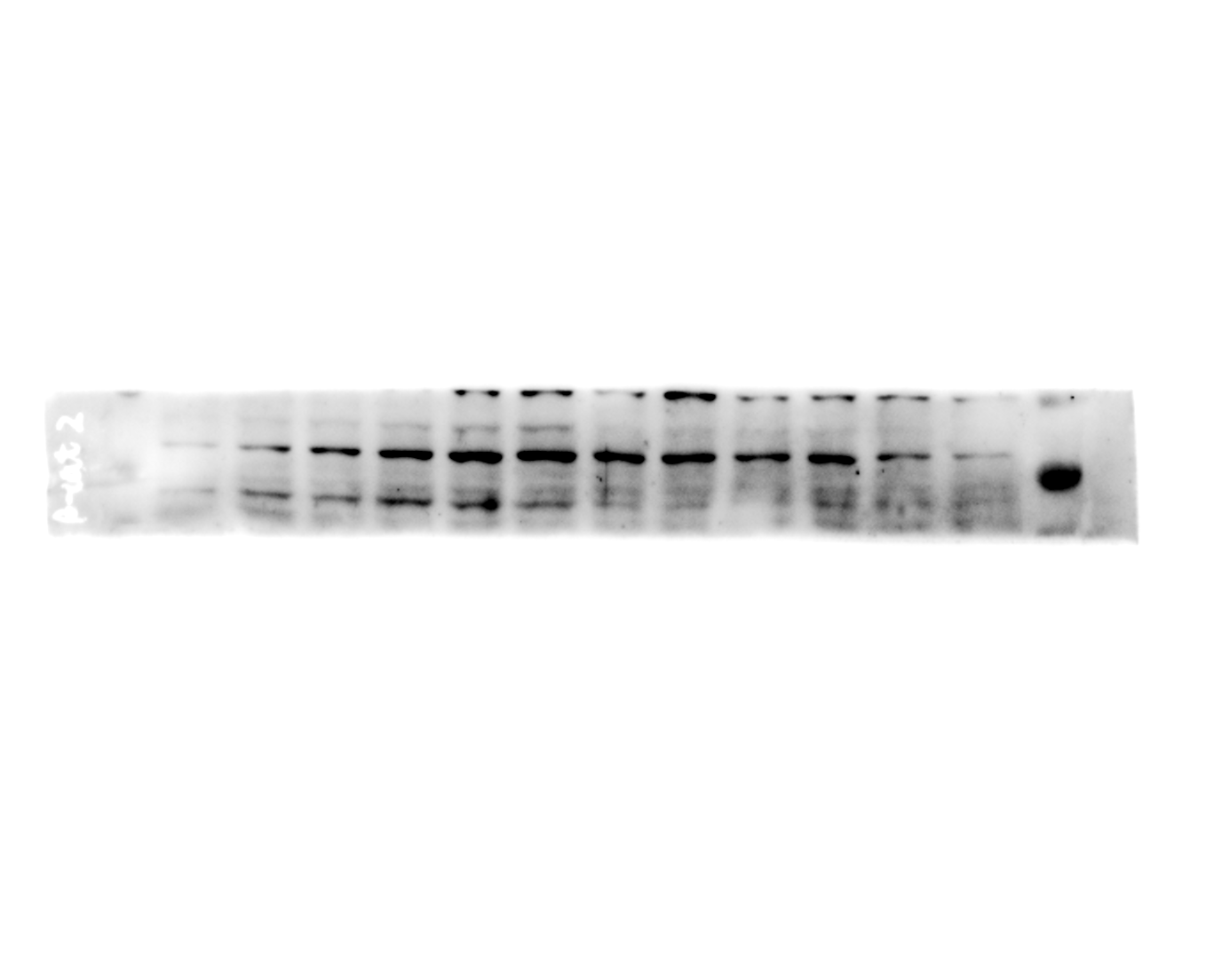


70KD

100KD

**Actin for Fig 4H**


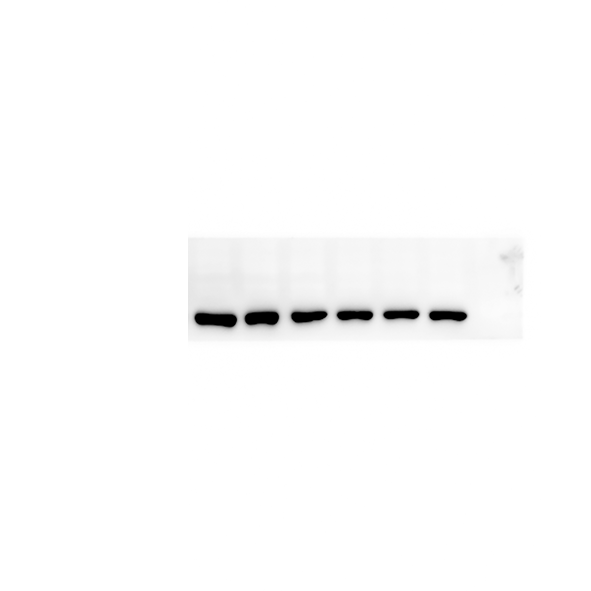


55KD

**Fibronectin** **for Fig 4H**


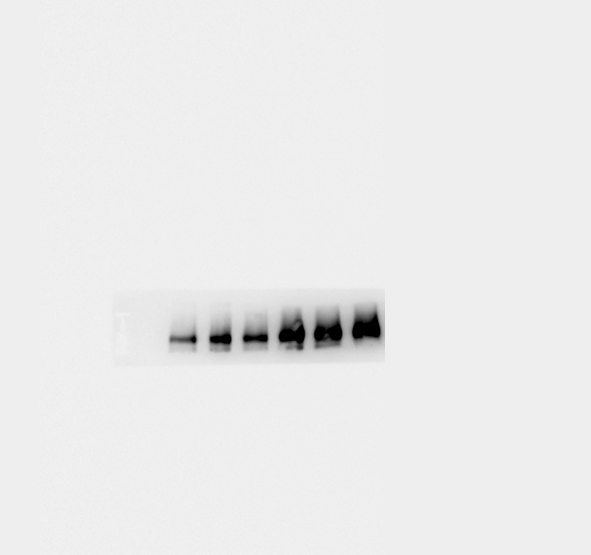


250KD

300KD

**Actin for Fig 4H**


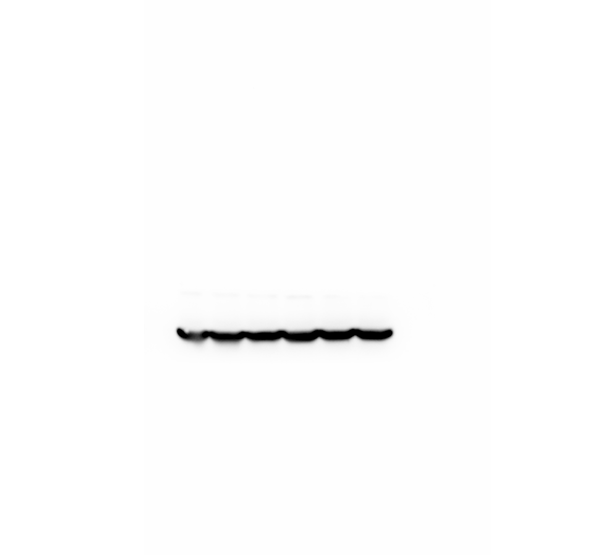


40KD

55KD

**β-catenin for Fig 4H**


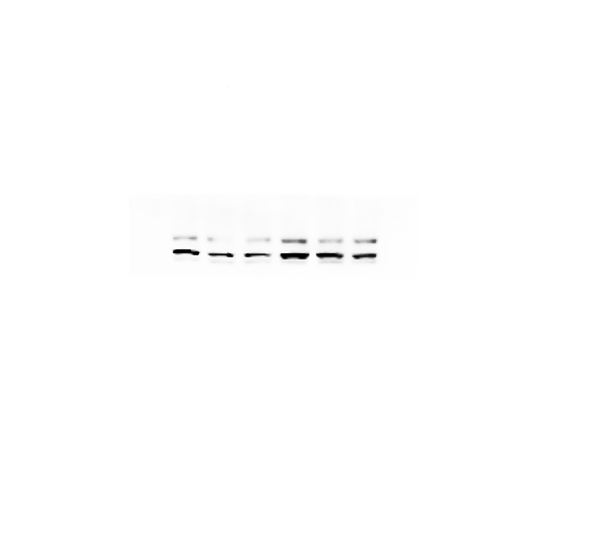


100KD

70KD

**Actin for Fig 4J**


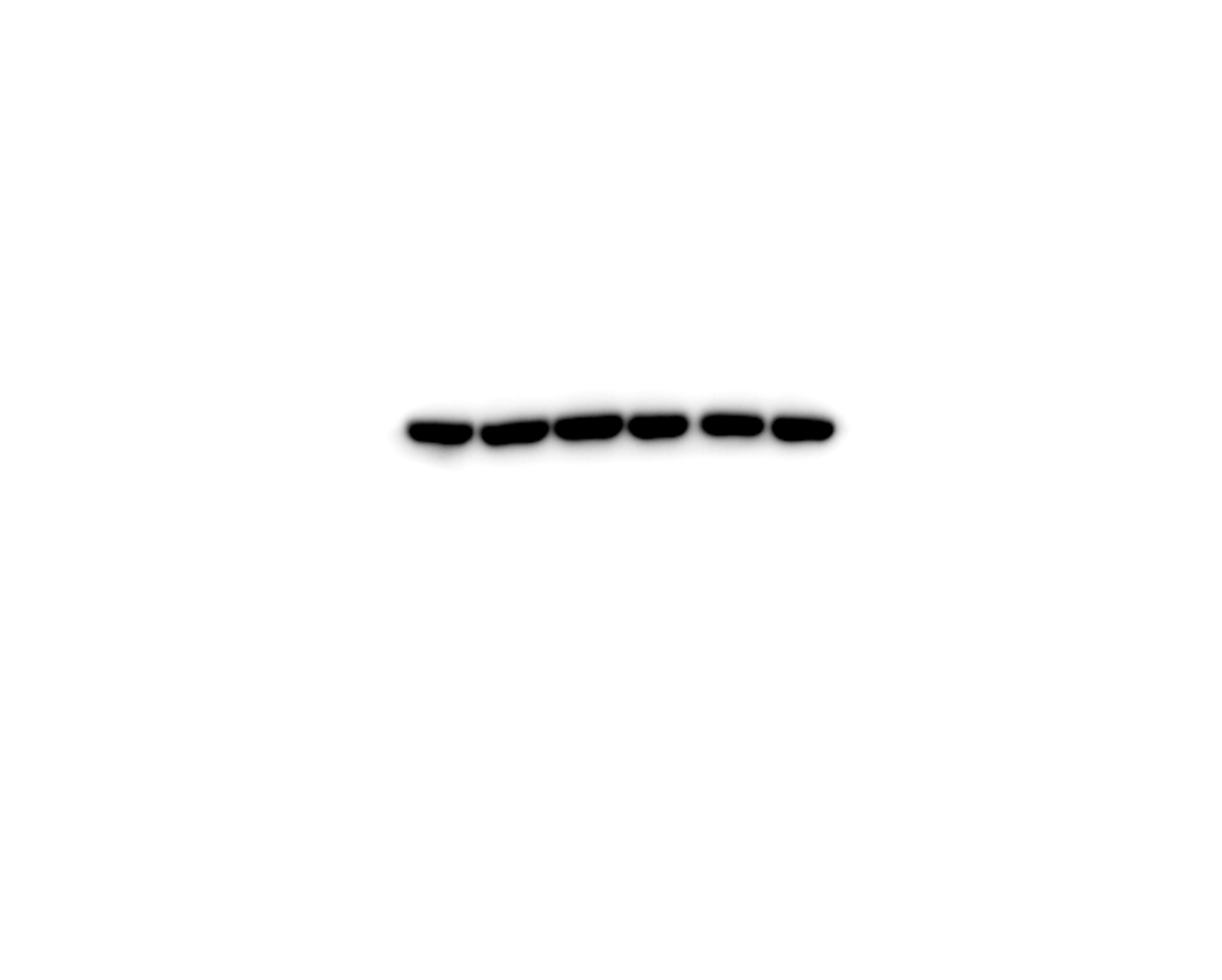


40KD

**sfrp5 for Fig 4J**


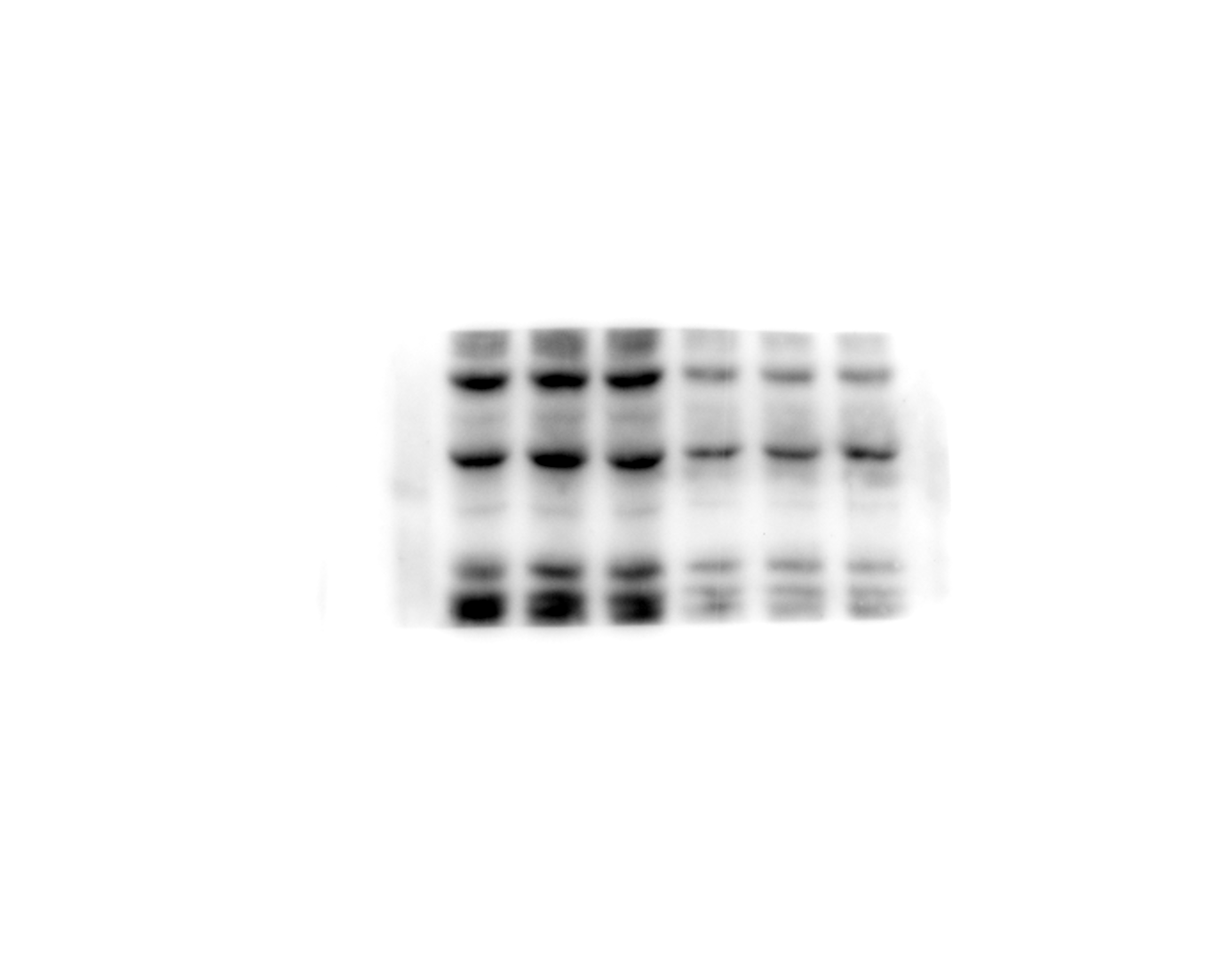


35KD

40KD

**Actin for Fig 5A**


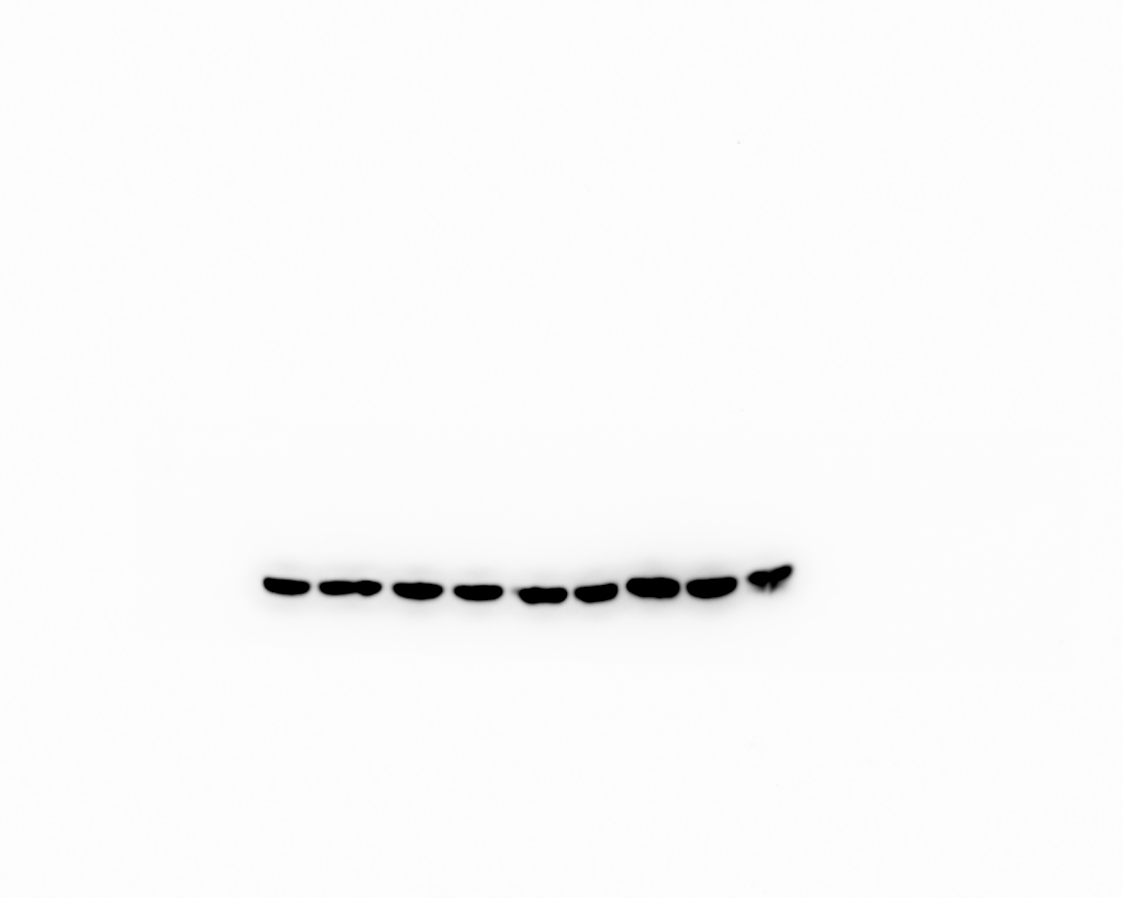


40D

**E-cadherin for Fig 5A**


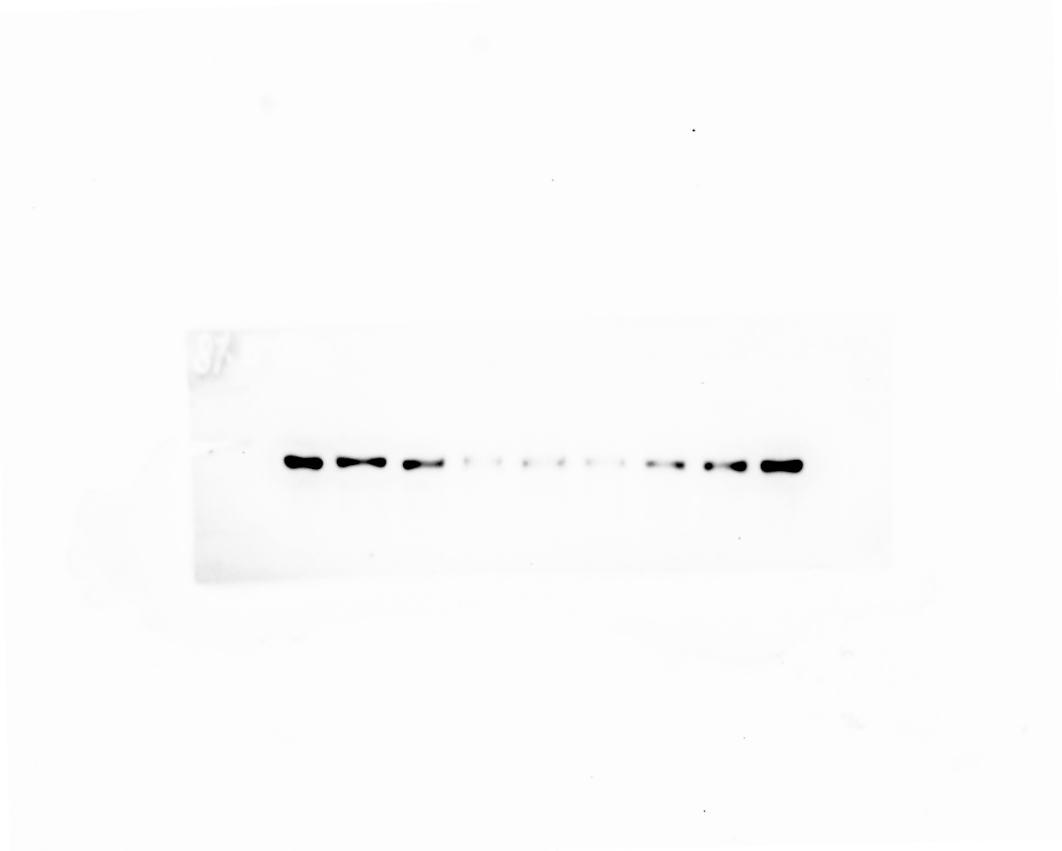


100KD

130KD

**Fibronectin for Fig 5A**


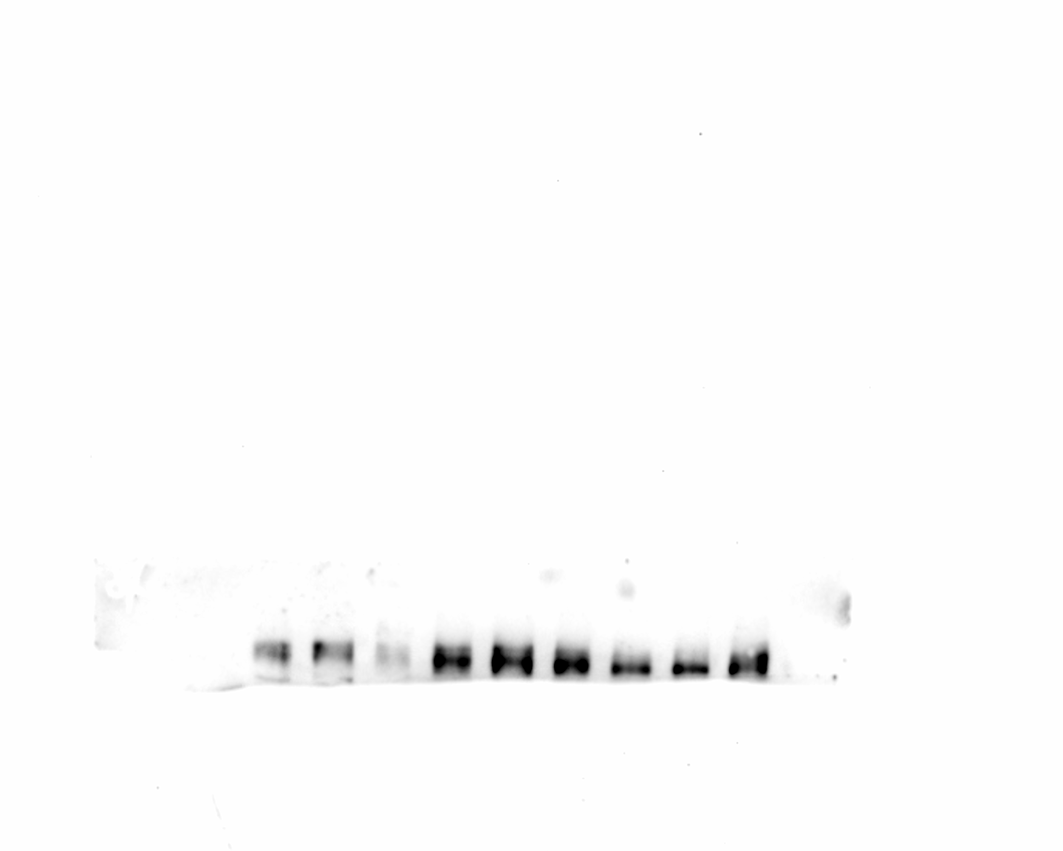


300KD

**GSK3β for Fig 5A**


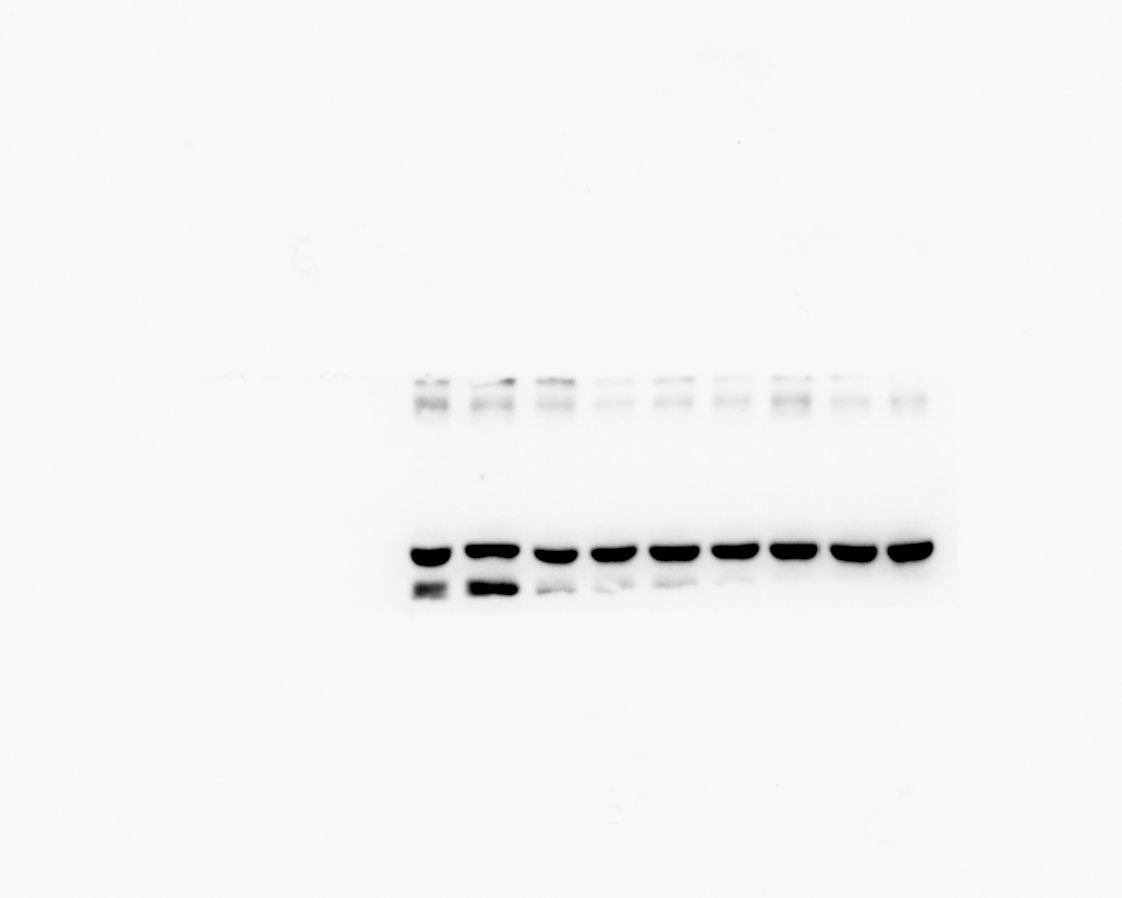


40KD

**p-GSK3β for Fig 5A**


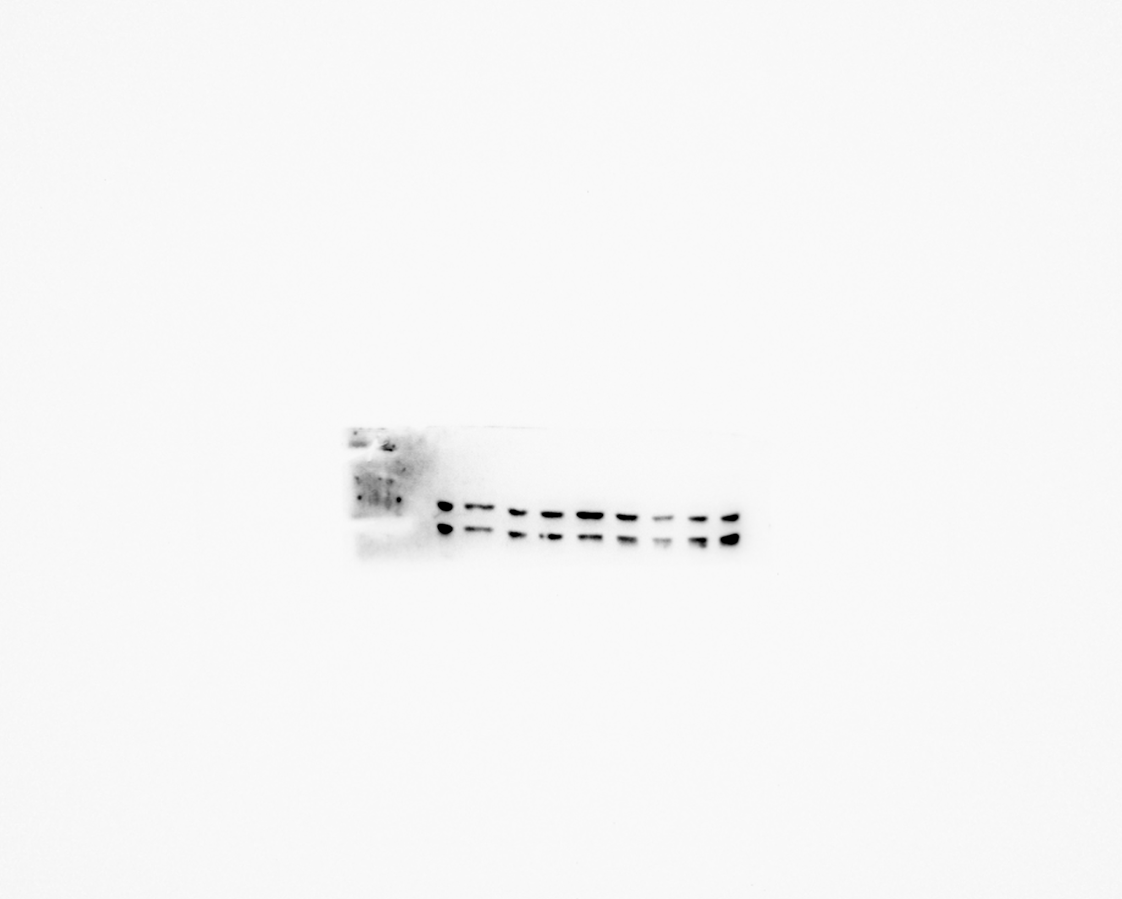


40KD

55KD

**β-catenin for Fig 5A**


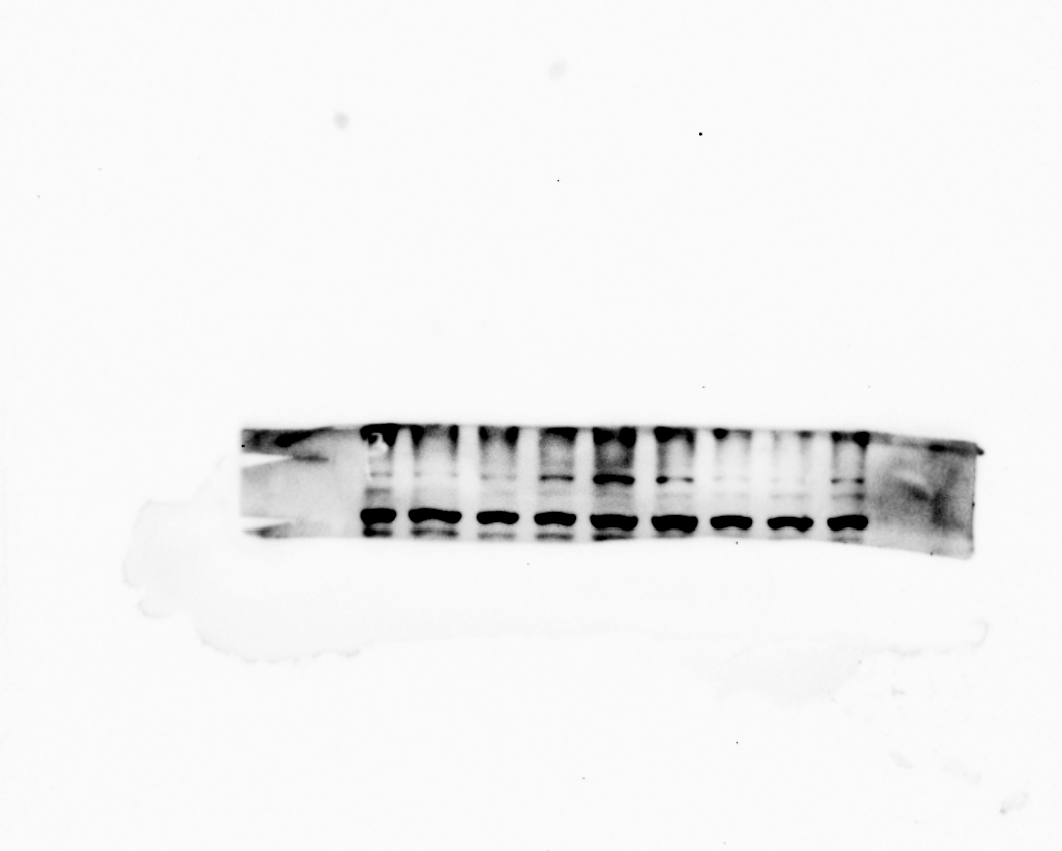


100KD
